# DNA methylation is associated with hair trace elements in female adolescents from two vulnerable populations in the Colombian Caribbean

**DOI:** 10.1093/eep/dvae008

**Published:** 2024-06-20

**Authors:** Alejandra Manjarres-Suarez, Anne Bozack, Andres Cardenas, Jesus Olivero-Verbel

**Affiliations:** Environmental and Computational Chemistry Group, School of Pharmaceutical Sciences, Zaragocilla Campus, University of Cartagena, Cartagena 130015, Colombia; Department of Epidemiology and Population Health, Stanford University, Stanford, CA 94305, United States; Department of Epidemiology and Population Health, Stanford University, Stanford, CA 94305, United States; Environmental and Computational Chemistry Group, School of Pharmaceutical Sciences, Zaragocilla Campus, University of Cartagena, Cartagena 130015, Colombia

**Keywords:** EWAS, differentially methylated positions, teenagers, chemical elements

## Abstract

Exposure to trace elements (TEs) influences DNA methylation patterns, which may be associated with disease development. Vulnerable populations, such as adolescents undergoing maturity, are susceptible to the effects of TE exposure. The aim of this study was to analyze the association of hair TE concentration with DNA methylation in a sample from female adolescents living in two communities in the Colombian Caribbean coast. Hair and blood samples were obtained from 45 females, between 13 and 16 years of age. Seventeen TEs were quantified in hair samples. DNA methylation was measured in leukocytes using the Infinium MethylationEPIC BeadChip. Linear models were employed to identify differentially methylated positions (DMPs) adjusting for age, body mass index, mother’s education, and cell type composition. Among the tested elements, vanadium, chromium, nickel, copper, zinc, yttrium, tin, and barium were significantly associated with DMPs (false discovery rate < 0.05), registering 225, 1, 2, 184, 1, 209 189, and 104 hits, respectively. Most of the DMPs were positively associated with TEs and located in open sea regions. The greatest number of DMPs was annotated to the *HOXA3* and *FOXO3* genes, related to regulation of gene expression and oxidative stress, respectively. These findings suggest that DNA methylation may be involved in linking exposure to TEs among female adolescents to downstream health risks.

## Introduction

The environment plays a key role in human well-being and quality of life. Environmental exposures influence health through molecular pathways including epigenetic mechanisms [1], which refers to heritable changes in gene expression that occur without changing the DNA sequence directly [2], with DNA methylation (DNAm) being the most studied, and it is influenced by numerous environmental factors, including exposure to pollutants, such as trace elements (TEs) [1, 3, 4].

Exposure to TEs occurs primarily through respiration or ingestion [5, 6]. Populations with high seafood intake, living in areas with polluted water [7, 8], and/or near industrial zones, or using chemicals containing TEs in their occupation [6, 9] may be exposed to high concentrations of TEs. TEs are classified as essential, likely essential, and nonessential or toxic [5]. Exposure to toxic TEs or abnormally low or high physiological concentrations of essential TEs can affect the neurological, cardiovascular, immune, hematological, reproductive, and urinary systems, increasing the risk for cancer and noncancer diseases [5, 6, 10–16]. The health risks of TE exposure may also vary by life stage and sex. For example, mixtures of metals have been associated with changes in sex hormones among female, but not male, children [17]. Exposure to lead (Pb) has also been associated with levels of reproductive hormones, with potential effect modification by cadmium [Cd, 18].

Perturbations in DNAm may be involved in pathways linking TE exposure to disease risk [3]. In addition, metal and metalloid exposure may alter DNAm by increasing reactive oxygen species (ROS), which in turn could interfere with the activity of methyltransferase enzymes [1]. Variations in DNAm associated with environmental exposures including TEs have been identified using epigenome-wide association studies (EWASs) [4, 19, 20]. EWAS is a hypothesis-free method that interrogates methylation loci across the epigenome [21]. EWAS commonly uses high-throughput platforms such as the Illumina Infinium MethylationEPIC BeadChip (850 K), which measures DNAm at >850 000 cytosine-guanine dinucleotides (CpG sites) [22].

Both essential TEs, such as copper (Cu) and selenium (Se), and nonessential TEs, such as arsenic (As), mercury (Hg), Cd, and Pb, have been associated with DNAm [2, 23–28]. The epigenome may be particularly sensitive to TE exposure during periods of developmental programming, resulting in perturbations that may impact health risks in adulthood [29]. Multiple EWAS has reported the impact of prenatal As exposure in cord blood and placenta samples [30–32]. Prenatal Hg [33], Pb [34], and other nonessential and essential TEs [4] have also been associated with DNAm in cord blood samples. However, studies of TE exposure during childhood or adolescence are lacking. In addition, vulnerable populations with precarious socioeconomic conditions, most specifically children and adolescents, are of concern [2].

The purpose of this study was to investigate the extent to which the concentrations of 17 TEs measured in hair were associated with leukocyte DNAm in a sample from female adolescents living in vulnerable conditions in the Colombian Caribbean coast, specifically, from Tierra Bomba Island (Cartagena, Bolivar) and Berrugas (San Onofre, Sucre). It is important to note that this study is a preliminary investigation and replicas are suggested.

## Materials and Methods

### Study population

This study involved 45 female adolescents, a subset of a full cohort of 202 adolescents. Participants were recruited from two coastal sites in northern Colombia, Tierra Bomba Island (Bolivar Department), and San Onofre (Sucre Department). Tierra Bomba Island is located near an industrial zone with pollutant sources, whereas San Onofre is a community lacking essential services such as municipal drinking water. Exposure to TEs among community members of Tierra Bomba Island occurs through industrial emissions and seafood sources [35]. The selection criteria were as follows: female, between 13 and 16 years of age, and having lived in the studied area for at least the past 10 years. This study focused on this population due to biological development that occurs during adolescence, which may affect susceptibility to exposure to xenobiotics, particularly among female children [17, 18].

The study was approved by the Ethics Committee of the University of Cartagena, Colombia (Act No. 97 of 2 June 2017). Written informed consent and assent were obtained from parents and participants, respectively.

### Sociodemographics

A sociodemographic questionnaire including basic information on the mother’s education level and anthropometric information was administered to all participants.

### Sample collection and processing

Hair and blood samples were obtained from sampling campaigns carried out during 2018 and early 2019. The collection, transport, and processing of the hair samples for TE analysis were carried out as previously described by Manjarres-Suarez and Olivero-Verbel [36] and Manjarres-Suarez *et al*. [35]. In brief, ∼20–200 mg of hair from the occipital region was obtained with sterile scissors and stored in paper envelopes until it was processed by the laboratory. For the analysis of TEs, hair from the opposite end to the cut site was used. In addition, 3 ml of blood was extracted in tubes coated with ethylenediaminetetraacetic acid anticoagulant for DNAm analysis. The blood samples were transported to the laboratory at 4°C and stored at −80°C until DNA extraction. DNA was extracted from buffy coat using the PureLink® Genomic DNA Mini kit (Invitrogen, Carlsbad, CA, USA), in accordance with the manufacturer’s recommendations. DNA concentration was quantified using a NanoDrop 2000 (Thermo Scientific), and the purity was evaluated by measuring the A260/A280 ratio.

### TE concentration

Concentrations of the following TEs were measured in hair samples: boron (B), scandium (Sc), vanadium (V), chromium (Cr), cobalt (Co), nickel (Ni), copper (Cu), zinc (Zn), arsenic (As), yttrium (Y), molybdenum (Mo), cadmium (Cd), tin (Sn), barium (Ba), tungsten (W), mercury (Hg), and lead (Pb). Mercury concentration was determined using an RA-915M Mercury Analyzer with a PYRO-915+ pyrolyzer. Calibration curves were determined with certified materials IAEA-085 and IAEA-086 from the International Atomic Energy Agency (Analytical Quality Control Services). All samples were measured in duplicate. The remaining TEs were quantified using an Agilent 7700 ICP-MS with calibration curves constructed using a series of standard solutions containing a mixture of all examined elements (1–250 ppb). Accuracy was assessed using Certified Reference Materials (ERM-DB 001, NIES 13, IAEA-085, IAEA-086, DORM-3). The limits of detection (LOD) and limits of quantification are shown in Supplementary Table S1. All measurements were above the LOD. Correlations between TEs were calculated using GraphPad Prism 6.

### DNA methylation assessment

DNAm was measured using the Infinium MethylationEPIC BeadChip (Illumina, San Diego, CA, USA). The analyses were conducted at the California Institute for Quantitative Biosciences, University of California, Berkeley. Samples were randomized to sample plates prior to measurement. All DNAm data were imported into R statistical software [37] and preprocessed using functional normalization in the “minfi” package [38]. ComBat [39] from the “sva” package was used to remove residual effects from the sample plate. For analysis, failed probes (detection *P* > .05), probes annotated to the X chromosome, cross-reactive probes [40], and single-nucleotide polymorphism (SNP)-associated probes (probes with a SNP at the single base extension, probes with polymorphic CpGs, and probes located with 10 base pairs of SNPs with minor allele frequencies ≥ 0.05) were excluded. A total of 717 885 autosomal CpG loci were included in analyses. The DNAm status for each CpG locus was calculated as a beta value, ranging from 0 to 1 representing percent methylation. Beta values were logit transformed to M-values for statistical analysis [41]. White blood cell composition was estimated from DNAm measurements using the Houseman projection method [42] implemented through the “estimateCellCounts” function in “minfi” [38].

### Statistical analyses

Descriptive statistics were calculated as the mean ± standard error of the mean (SEM) for continuous variables and percentages for categorical variables. Hair TE concentrations were log2 transformed to meet model assumptions due to right-skewed data. EWAS studies may be confounded by cellular heterogeneity [43]. Therefore, to understand if TE concentrations were associated with cell type composition, simple linear regression models were used to evaluate associations of each TE in hair and estimated cell type proportions.

Associations of log2-transformed hair TE concentrations and DNAm were analyzed using the R package “limma.” Each TE was analyzed separately. Models were adjusted for age, body mass index (BMI), maternal education level, and estimated cell type composition. It is important to note that since EWAS studies could present complications for cellular heterogeneity, white blood cell composition is adjusted for [44]. These covariates were selected due to previously reported associations with DNAm [42, 44–46]. By study design, hair TE concentrations differed between study sites [33, 34], and therefore we did not include study site as a confounder. Analyses were adjusted for multiple comparisons using the Benjamini and Hochberg false discovery rate (FDR) correction [47] and the Bonferroni correction using the “p.adjust” function. Probes were considered significant at a cutoff of FDR < 0.05. CpG annotations were obtained from the “IlluminaHumanMethylationEPICanno.ilm10b4.hg19” annotation package. Results are presented as the number of associations (hits) and the distribution of hits relative to CpG islands. Effect estimates for each TE evaluated are reported as the log fold change (FC). EWAS were performed using R, version 4.0.5 [37].

## Results

### Population characteristics

Participant characteristics are shown in Table 1. All participants were female and Afro-descendants between 13 and 16 years old who lived in two coastal sites in northern Colombia, Tierra Bomba Island (Bolivar Department), and San Onofre (Sucre Department). Most participants (77.8%) had mothers with some secondary education.

**Table 1.** Characteristics of the study population

Variable	Categories	*n* (%)
Area	Tierra Bomba Island	23 (51.1)
	San Onofre	22 (48.9)
Age	Years^a^	14.84 ± 0.12
Anthropometry^b^	Height (m)	1.59
	Weight (kg)	54.1
	BMI (kg/m)	21.41 ± 0.59
Mother’s education	Some primary school	10 (22.2)
	Some secondary school	35 (77.8)

*n*, number of participants.

a Results presented as mean ± SEM.

b Results are shown as mean and, for BMI, average ± SEM.

### TE concentrations

Descriptive statistics for the TE levels are presented in Table 2. For Hg, the mean value (1.32 µg/g) was greater than the maximum allowable concentration set by the US Environmental Protection Agency (1 µg/g) [48].

**Table 2.** Descriptive statistics for TEs evaluated in this study

TE	Mean ± SEM (µg/g)	Median (µg/g)	Interquartile range (25–75%)
B	1.36 ± 0.16	1.10	0.75–1.64
Sc	0.01 ± 0.001	0.01	0.01–0.01
V	0.11 ± 0.01	0.09	0.07–0.15
Cr	11.3 ± 2.32	5.18	3.39–15.4
Co	0.04 ± 0.01	0.03	0.02–0.04
Ni	0.50 ± 0.16	0.27	0.18–0.41
Cu	8.31 ± 0.76	6.96	5.63–9.77
Zn	45.3 ± 4.53	35.3	24.2–60.2
As	1.83 ± 0.62	0.66	0.22–1.54
Y	0.01 ± 0.001	0.01	0.01–0.01
Mo	0.06 ± 0.005	0.05	0.04–0.07
Cd	0.03 ± 0.003	0.02	0.01–0.03
Sn	0.95 ± 0.09	0.92	0.52–1.21
Ba	1.76 ± 0.20	1.52	0.77–2.43
W	0.01 ± 0.002	0.01	0.00–0.01
Hg	1.32 ± 0.15	1.01	0.58–1.81
Pb	1.01 ± 0.21	0.57	0.32–1.09

Spearman correlations were calculated out between the TEs evaluated. With the exception of Cr, all TEs significantly correlated with at least one other TE (*P* < .05; Supplementary Table S2). The greatest correlations were Co:V (*ρ* = 0.73; *P* < .001), Co:Y (*ρ* = 0.67; *P* < .001), Cu:Ni (*ρ* = 0.61; *P* < .001), Pb:Cd (*ρ* = 0.66; *P* < .001), and Pb:Mo (*ρ* = 0.65; *P* < .001).

Associations of TE hair concentrations with estimated white blood cell proportions were evaluated using simple linear regression models (Supplementary Table S3). There were significant positive associations for Co and monocytes (*B* = 17.64; *P *= .004), Cu and CD8+ T lymphocytes (*B* = 8.31; *P *= .046), and Cd with both CD8+ T lymphocytes (*B* = 9.41; *P *= 0.029) and monocytes (*B* = 12.89; *P* = .046).

### TE and DNAm associations

Eight of 17 TEs evaluated were significantly associated with at least one differentially methylated position (DMP) (FDR < 0.05). Table 3 summarizes the findings. For most elements, the genomic inflation factor was approximately 1, indicating the goodness of fit of the models [49]. The *Q*–*Q* plots are shown in Supplementary Fig. S1. Volcano plots and Manhattan plots for each of the eight TEs with significant DMPs are shown in Fig. 1.

**Table 3.** Summary of numbers of DMPs and genomic inflation factors for the hair concentrations of TEs.

TE	*λ* ^a^	FDR < 0.05	*P*_Bonferroni_ < .05
V	1.02	225	55
Cr	1.57	1	1
Ni	1.03	2	1
Cu	0.92	184	21
Zn	1.07	1	1
Y	0.91	209	16
Sn	0.80	189	20
Ba	0.98	104	6

Models adjusted for age, BMI, maternal education, and estimated cell type composition. TEs not associated with any DMPs are not shown.

a Genomic inflation factor.

**Figure 1.**
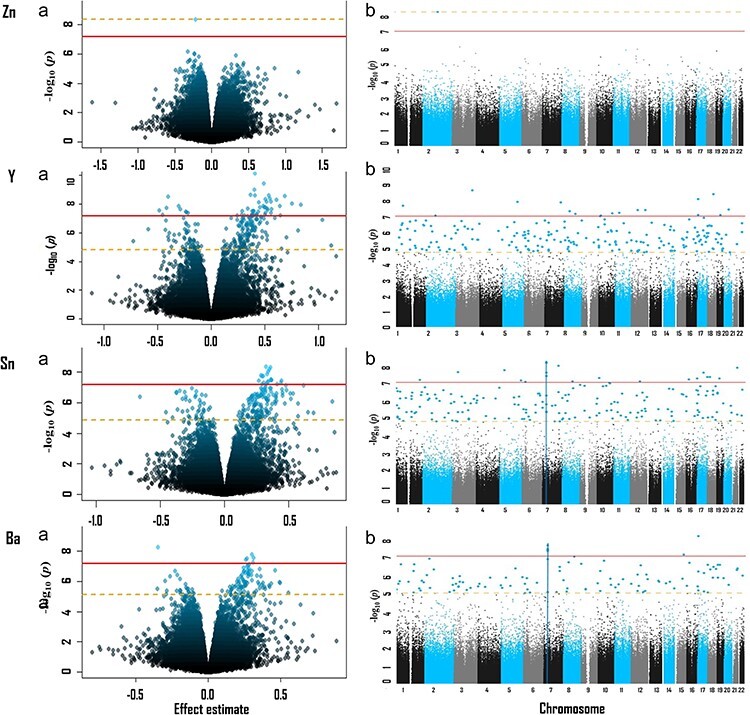
Volcano (a) and Manhattan (b) plots for the EWAS hair concentrations of TEs with significant DMPs (FDR < 0.05). The Bonferroni and FDR levels of significance (<0.05) are indicated by a solid and dashed line, respectively. The blue vertical lines in Manhattan plots for Sn and Ba indicate genes containing multiple DMPs. Models adjusted for age, BMI, maternal education, and estimated cell type proportions.

**Figure 1.**
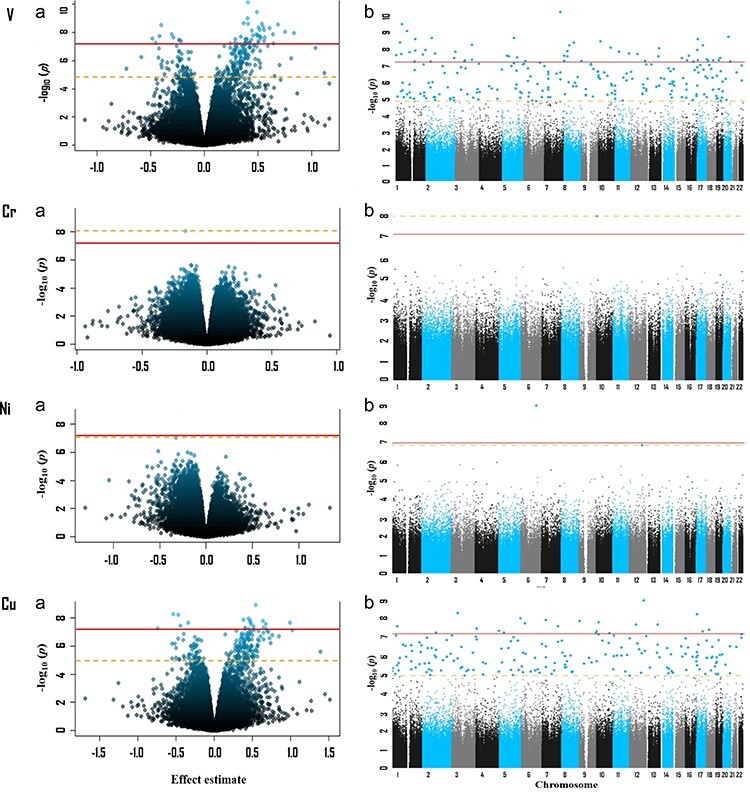
(Continued)

Cr was associated with one DMP, cg09139549, annotated to *PFKP* (chromosome 10, log FC = −0.16; *P* = 8.98 × 10^−9^). Zn was associated with the DMP cg13686662 (chromosome 2, log FC = −0.22, *P* = 4.39 × 10^−9^). Ni was associated with two DMPs, cg16689243 (chromosome 6, log FC = −0.25, *P* = 5.91 ×10^−10^) and cg26591794 (chromosome 12, log FC = −0.33, *P* = 8.93 × 10^−8^). All these associations were located in the open sea area.

The top results for analyses of the other TEs are summarized Tables 4–8; all significant DMPs (FDR < 0.05) are included in Supplementary Tables S4–S8. V was associated with 225 DMPs (Table 4 and Supplementary Table S4). Effect estimates were mostly positive (72.4%), and most associations were in open sea regions (53.3%).

**Table 4.** Top 10 DMPs associated with V adjusted for age, BMI, mother education, and estimated cell type proportions

*P*-value	Chr	Pos^a^	CpG	Log FC	FDR	Relation to island	Gene
7.34 × 10^−11^	7	128 697 416	cg06396390	0.41	<0.001	S_Shore	
3.8 × 10^−10^	1	50 883 329	cg22711792	0.49	<0.001	Island	*DMRTA2*
1.02 × 10^−9^	1	91 192 798	cg14663264	0.40	0.001	Island	
2.21 × 10^−9^	20	36 628 932	cg01720774	0.37	0.002	OpenSea	*TTI1*
2.49 × 10^−9^	5	93 954 434	cg11339965	0.41	0.002	Island	*ANKRD32*
2.53 × 10^−9^	2	45 870 883	cg08715805	0.54	0.002	Island	
2.91 × 10^−9^	1	161 981 053	cg27316795	0.69	0.002	OpenSea	*OLFML2B*
3.03 × 10^−9^	17	3 704 574	cg13984928	−0.40	0.002	OpenSea	*ITGAE*
4.17 × 10^−9^	10	17 051 623	cg20434599	0.55	0.003	OpenSea	*CUBN*
4.69 × 10^−9^	8	81 398 656	cg22380165	0.51	0.003	Island	*ZBTB10*

Abbreviations: Chr, chromosome; Pos, position.

a hg19 assembly.

Cu was associated with 184 DMPs (FDR < 0.05) (Table 5 and Supplementary Table S5). Effect estimates were mostly positive (72.3%). Most DMPs were the open sea regions (59.8%), followed by CpG islands, shore, and shelf regions.

**Table 5.** Top 10 DMPs associated with Cu adjusted for age, BMI, mother education, and estimated cell type proportions

*P*-value	Chr	Pos^a^	CpG	Log FC	FDR	Relation to island	Gene
1.21 × 10^−9^	12	113 440 283	cg24531077	0.54	0.001	OpenSea	*OAS2*
5.79 × 10^−9^	3	47 563 649	cg04507495	−0.54	0.002	OpenSea	
6.37 × 10^−9^	17	3 704 574	cg13984928	−0.47	0.002	OpenSea	*ITGAE*
1.08 × 10^−8^	5	172 566 090	cg25152909	0.45	0.002	OpenSea	*C5orf41*
1.32 × 10^−8^	7	27 154 914	cg00318947	0.38	0.002	N_Shore	*HOXA3*
1.52 × 10^−8^	8	144 775 130	cg05342816	0.47	0.002	N_Shore	*ZNF707*
1.75 × 10^−8^	10	17 051 623	cg20434599	0.64	0.002	OpenSea	*CUBN*
2.03 × 10^−8^	6	29 442 830	cg10619365	0.47	0.002	OpenSea	
2.23 × 10^−8^	13	97 422 029	cg09122588	0.99	0.002	OpenSea	*HS6ST3*
2.29 × 10^−8^	12	51 419 973	cg04118903	−0.34	0.002	Island	*SLC11A2*

Abbreviations: Chr, chromosome; Pos, position.

a hg19 assembly.

Similarly, Y was associated with 209 DMPs with largely positive effect estimates (Table 6 and Supplementary Table S6). DMPs were mostly located in open sea regions, followed by CpG islands and shore regions, with the lowest percentage in shelf regions.

**Table 6.** Top 10 DMPs associated with Y adjusted for age, BMI, mother education, and estimated cell type proportions

*P*-value	Chr	Pos^a^	CpG	Log FC	FDR	Relation to island	Gene
3.84 × 10^−11^	13	90 045 847	cg24712244	−0.27	0.000	OpenSea	
1.58 × 10^−9^	3	138 830 878	cg14638147	0.41	0.001	OpenSea	*BPESC1*
2.71 × 10^−9^	18	47 123 950	cg12490577	0.42	0.001	OpenSea	
5.78 × 10^−9^	17	3 704 574	cg13984928	−0.40	0.001	OpenSea	*ITGAE*
8.29 × 10^−9^	5	122 947 164	cg03722513	−0.24	0.001	OpenSea	*CSNK1G3*
9.41 × 10^−9^	7	128 697 416	cg06396390	0.38	0.001	S_Shore	
1.50 × 10^−8^	1	53 207 187	cg09509462	−0.34	0.002	OpenSea	*ZYG11B*
2.56 × 10^−8^	20	36 628 932	cg01720774	0.36	0.002	OpenSea	*TTI1*
2.77 × 10^−8^	12	113 440 283	cg24531077	0.44	0.002	OpenSea	*OAS2*
2.84 × 10^−8^	12	51 419 973	cg04118903	−0.29	0.002	Island	*SLC11A2*

Abbreviations: Chr, chromosome; Pos, position.

a hg19 assembly.

Sn was associated with 189 DMPs (Table 7 and Supplementary Table S7). All CpGs below the Bonferroni cutoff (adjusted *P* < .05) had positive effect estimates. Although DMPs were distributed on all chromosomes, many of the most significant DMPs were located in chromosome 7 (vertical blue line in Manhattan plot of Fig. 1 for Sn) and a majority of them were associated with *HOXA3* gene. DMPs were most commonly located in open sea regions, followed by CpG islands, shore regions, and shelf regions.

**Table 7.** Top 10 DMPs associated with Sn adjusted for age, BMI, mother education and estimated cell type proportions

*P*-value	Chr	Pos^a^	CpG	Log FC	FDR	Relation to island	Gene
4.69 × 10^−9^	7	27 155 173	cg04351734	0.32	0.002	Island	*HOXA3*
4.87 × 10^−9^	7	27 155 036	cg16406967	0.36	0.002	Island	*HOXA3*
7.11 × 10^−9^	7	128 415 071	cg09658621	0.35	0.002	OpenSea	*OPN1SW*
9.24 × 10^−9^	21	43 324 038	cg02590715	0.34	0.002	N_Shelf	*C2CD2*
1.33 × 10^−8^	5	39 373 418	cg02653030	0.33	0.002	OpenSea	*DAB2*
1.73 × 10^−8^	3	44 912 362	cg02449689	0.29	0.002	OpenSea	
1.81 × 10^−8^	17	49 450 892	cg02960148	0.29	0.002	OpenSea	
1.82 × 10^−8^	7	27 155 002	cg16748008	0.30	0.002	Island	*HOXA3*
2.96 × 10^−8^	7	27 155 039	cg18680977	0.36	0.002	Island	*HOXA3*
3.26 × 10^−8^	18	19 799 634	cg15651884	0.37	0.002	OpenSea	

Abbreviations: Chr, chromosome; Pos, position.

a hg19 assembly.

Results for Ba (Table 8 and Supplementary Table S8) had similar patterns to those of Sn. Most DMPs had positive effect estimates. Although DMPs were distributed on all chromosomes, several DMPs were also located on chromosome 7 (vertical blue line in Manhattan plot of Fig. 1 for Ba) and most of them were also associated with *HOXA3* gene. Among the DMPs, 59.6% were in open sea regions and 14.4% were in CpG islands.

**Table 8.** Top 10 DMPs associated with Ba adjusted for age, BMI, mother education, and estimated cell type proportions

*P*-value	Chr	Pos^a^	CpG	Log FC	FDR	Relation to island	Gene
5.55 × 10^−9^	17	2 652 918	cg09652807	−0.35	0.004	Island	*MIR1253*
1.76 × 10^−8^	7	27 155 036	cg16406967	0.30	0.005	Island	*HOXA3*
2.80 × 10^−8^	7	27 155 039	cg18680977	0.31	0.005	Island	*HOXA3*
3.21 × 10^−8^	7	27 155 173	cg04351734	0.27	0.005	Island	*HOXA3*
3.72 × 10^−8^	7	27 155 002	cg16748008	0.26	0.005	Island	*HOXA3*
5.54 × 10^−8^	15	76 151 117	cg11384517	0.31	0.007	OpenSea	*UBE2Q2*
9.51 × 10^−8^	2	38 831 686	cg02565468	0.27	0.008	S_Shore	
1.05 × 10^−7^	7	27 154 845	cg16644023	0.27	0.008	N_Shore	*HOXA3*
1.23 × 10^−7^	10	47 667 303	cg26231261	0.25	0.009	OpenSea	*ANTXRL*
1.58 × 10^−7^	16	19 890 592	cg01620602	0.28	0.009	OpenSea	*GPRC5B*

Abbreviations: Chr, chromosome; Pos, position.

a hg19 assembly.

Overall, hair concentrations of TEs were positively associated with methylation levels of DMPs. The *HOXA3* and *FOXO3* genes contained the highest number of significant DMPs, associated with V, Cu, Y, Sn, and Ba, in all cases with positive effects. DMPs were also annotated to members of the superfamily *ZNF*, including *ZNF133, ZNF536, ZNF642, ZNF704*, and *ZNF707*, although only *ZNF707*, which also reported a positive effect, was identified for multiple elements (V, Cu, Y, Sn, and Ba).

**Figure 2.**
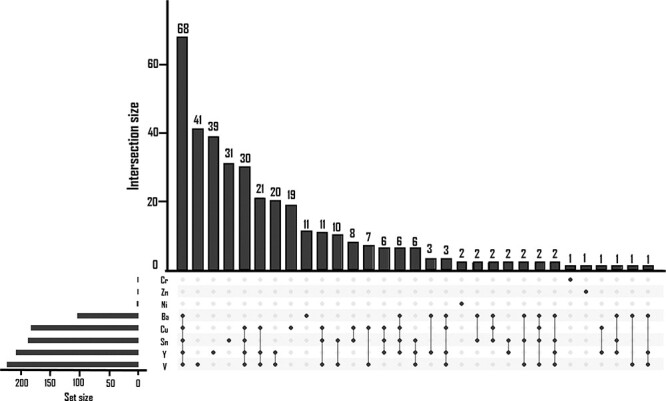
Upset plot of overlap between DMPs associated with hair concentrations of individual TEs (FDR < 0.05).

There was a substantial overlap between DMPs associated with hair concentrations of individual TEs, as can be seen in Fig. 2. In particular, Ba, Cu, Sn, Y, and V were associated with 68 common DMPs; Cu, Sn, Y, and V were associated with 30 common DMPs; Cu, Y, and V were associated with 21 common DMPs; and Y and V were associated with 20 common DMPs. Many TEs with common DMPs were significantly and positively correlated (e.g. Ba:Sn: *ρ* = 0.39; Ba:Y: *ρ* = 0.49; Cu:Y: *ρ *= 0.38; Cu:V: *ρ* = 0.48; Y:V: *ρ *= 0.50; *P *< .05; Supplementary Table S2).

## Discussion

This study reports epigenome-wide associations for hair TE concentrations in female adolescents from two populations of the Colombian Caribbean coast. Analyses of associations of TE concentrations with hematological parameters and gene expression in this population have been previously reported [35, 36]. In the current analyses, V, Cr, Ni, Cu, Zn, Y, Sn, and Ba showed associations with at least one differentially methylated CpG site. Cr, Zn, and Ni were each associated with 1–2 DMPs, while V (225 DMPs), Cu (184 DMPs), Y (209 DMPs), Sn (189 DMPs), and Ba (104 DMPs) were associated with the greatest number of DMPs. Overall, the highest proportion of DMPs were positively associated with hair TE concentrations and were located in open sea regions. To date, most studies carried out to determine the impact of TEs on epigenetics in humans have focused on metalloids and metals such as As, Hg, and Pb [8, 30, 50–57], elements that did not show associations with DNAm on this population, probably due to the low number of samples and exposure levels, or focus on female adolescents. To our knowledge, there are no previous EWASs of Y and Sn exposure; however, according to our data, these elements may be associated with differential DNAm and need to be considered in future studies.

Studies of associations of Cr, Zn, and Ni with DNAm have primarily been conducted *in vitro* or in animals. Cr has previously been associated DNAm. Global DNA hypomethylation has been associated with Cr in human cell lines, where cell cycle arrest was observed [58]. Site-specific DNA hypermethylation has been reported for *p16* evaluated in human bronchial epithelial cells after exposure to potassium dichromate [59], as well as in the *TBL1Y, FZD5*, and *IKZF2* genes of B-cell lymphoblasts exposed to soluble chromate [60]. In addition, an increase in methylation of a human bronchial epithelial cell line (16HBE) cells after exposure to Cr(VI) and in DNA repair genes in occupationally exposed workers was reported [61]. In this study, Cr was negatively associated with DNAm of *PFKP*, a gene that codes for a platelet isoform of phosphofructokinase 1, involved in glycolysis regulation and previously reported to promote cell proliferation [62]. Increases in global DNAm with Zn exposure have been reported in fish liver [63] and in humans [64]. For specific loci, negative associations were reported between Zn and CpGs of the *PARP1* gene [64]. A negative association between Zn and DNAm was also observed in this study for a CpG in chromosome 2 (cg13686662). There are limited data available describing the association of Ni with DNAm [65–67]; however, hypomethylation was observed in 16HBE cells treated with crystalline nickel sulfate [68].

Exposure to V has been investigated in gene-specific studies of DNAm in children, which have found differential DNAm in allergic and proinflammatory asthma genes among children in New York City [69], as well as in DNA repair genes among children in Mexico City [70]. The results of the current study showed an association between V and *ILF2* (Supplementary Table S4), a gene that has been implicated in inflammatory response [71].

Positive and negative associations of Cu with DNAm have previously been reported, which is in agreement with this study. Cu concentrations and placental DNAm in two cohorts in the USA were studied, and hypo- and hypermethylation were observed [72]. Positive associations between Cu and CpG sites of *NRF2* have been found in the umbilical cord blood of newborns from Mexico City [64]. However, greater high plasma Cu levels were associated with lower levels of DNAm in multiple genes in a genome-wide meta-analysis in a Chinese population [25].

The results found in the current study for Ba exposure contrast with previous studies. No associations were found between prenatal Ba exposure and DNAm in cord blood in a US-based cohort [4]. However, our findings suggest that hair Ba concentrations are associated with DNAm in female adolescents.

Genes with the greatest number of associations with hair TE concentrations in this study were *HOXA3* and *FOXO3*. The homeobox protein Hox-A3 (*HOXA3*) gene codes for a HOX transcription factor, which regulates gene expression during embryonic development and participates in the endothelial cell migration [73]. High levels in DNAm of this gene have been related to Alzheimer’s disease, Down syndrome’s brain development, and lung adenocarcinoma [74–76]. Results here showed that *HOXA3* had greater DNAm with increasing V, Cu, Y, Sn, and Ba concentrations. Forkhead box O3 (*FOXO3*) is a protein coding gene and is part of the subclass of forkhead transcription factors. It is known to participate in metabolic and oxidative stress and in the insulin/insulin-like growth factor signaling pathway. Additionally, this gene has been related to aging and longevity and plays an important role in the control of the cell cycle and apoptosis [77–81]. *FOXO3* methylation has been associated with a reduction in neuronal apoptosis induced by oxidative stress in cerebellar granular neurons [82]. *FOXO3* hypermethylation is fundamental in the pathophysiology of several cancers, and it has been related to the progression of myelodysplastic syndromes [83]. Increases in DNAm of *FOXO3* were also reported in this study with V, Cu, Y, Sn, and Ba exposure.

This study also identified positive and negative associations with DNAm at probes annotated to the zinc finger superfamily (*ZNF*) of genes. *ZNF133* and *ZNF536* showed negative associations with V, Cu, and Y. *ZNF133* is a protein coding gene and is involved in enabling DNA-binding transcription repressor activity [84], while *ZNF536* plays an important role in maintenance of neuronal cells as a negative regulator of neuron differentiation [85]. *ZNF707*, which had greater methylation with higher V, Cu, Y, Sn, and Ba exposure, may be involved in regulation of transcription by RNA polymerase II [86]. In addition, *ZNF707* has been associated with hypermethylation in research of endometriomas [87], which may be particularly relevant in this population of female adolescents.

More studies are required to understand the mechanisms linking TE exposure to DNAm. Oxidative damage may affect the ability of methyltransferases to interact with DNA [88]. For example, it has been proposed that when Cr(VI) enters cells, it is reduced to Cr(III) by nonenzymatic agents, generating ROS. Oxidation of a guanine to form 8-hydroxydeoxyguanosine could prevent DNA methyltransferase (DNMT) from a methylated neighboring cytosine [89–92]. The presence of ROS may also increase the activity of superoxide dismutase, which can deprotonate cytosine at the C-5 position and generate methylated cytosine through a reaction of DNA with positively charged S-adenosyl-l-methionine [91, 93]. Other proposed mechanisms include noncompetitive inhibition of the DNMT which interferes with catalytic activity [94].

The strengths of this study include an understudied population of female adolescents in Caribbean communities who may be particularly susceptible to the effects of environmental toxicants including TEs. This study used statistical methods to minimize bias and confounding, such as normalization of data and analysis with correction including the BMI, maternal education, and white blood cell composition. Future studies with greater power may explore cell type–specific differential DNAm using *in silico* methods [95]. A main limitation in this study was the small sample size, which limited power. The study was also restricted to female adolescents within a short age range, which did not allow analysis of the association between TE exposure and DNAm across ages and genders. Available data were limited to hair TE concentrations measured and leukocyte DNAm. Although the exposure and outcome were evaluated in different biological matrixes, we aimed to select tissues that would be the most relevant for the study objectives. Hair samples have advantages as a matrix for exposure biomarker assessment due to noninvasive sampling and representation on long-term exposure [96]. Although it has been demonstrated that hair concentrations of As, Mo, and Co correlate with concentrations measured in blood, urine, or water, hair concentration may not accurately reflect the internal dose for all TEs included in this study [97]. DNA methylation was measured in leukocytes, which may not be the biologically relevant target tissue for all phenotypic end points associated with TE exposure. However, blood has been shown to be a relevant surrogate tissue for studying DNAm of some genomic regions in adipose tissue [98], mammary tissue [99], and brain [100], and DNAm in target tissue may be estimated from levels measured in blood [101]. Furthermore, use of leukocyte DNAm in this study will allow for findings to be more readily replicated or compared to other epidemiological studies that commonly rely on DNAm measured in blood. Our study was also limited by analysis of associations between individual TEs and DNAm. Many of the TEs positively correlated and associated with common DMPs. Further research is needed to investigate the effects of mixtures of TEs and to understand if observed associations are being driven exposure to single TEs.

## Conclusion

This study is the first to investigate associations of hair TE concentrations with leukocyte DNAm in a population of female adolescents in the Colombian Caribbean. Analyses identified DMPs associated with essential, probably essential, and possibly toxic elements. Most DMPs were annotated to genes related to DNA-binding transcription factors and noncovalent interactions with nucleic acids. These findings suggest that perturbations in DNAm may be involved in pathways linking long-term TE exposure to health, particularly among female adolescents. However, this preliminary research warrants replication in larger studies in more diverse populations, and longitudinal studies are needed to understand the relationship between TE-associated DMPs and downstream health outcomes.

## Supplementary Material

dvae008_Supp

## Data Availability

All data generated in this work can be obtained under request.
