# Supplementary material for: DNA methylation is associated with hair trace elements in female adolescents from two vulnerable populations in the Colombian Caribbean
Source: Environ Epigenet. 2024 Jun 20;10(1):dvae008. doi: 10.1093/eep/dvae008 (PMC11548963; doi:10.1093/eep/dvae008)
Supplement: dvae008_Supp [file dvae008_supp.zip › Supplementary Material_Paper_EWAS_R2_VF.pdf]

DNA methylation is associated with hair trace elements in female adolescents from two vulnerable populations in the Colombian Caribbean

Supplementary Material

Table S1. Limits of detection (LOD) and limits of quantification (LOQ) for trace element analysis in human hair.

Trace element	LOD (μg/g)	LOQ (μg/g)
**B**	0.021	0.06
**Sc**	0.002	0.005
**V**	0.002	0.01
**Cr**	0.017	0.05
**Co**	0.0004	0.001
**Ni**	0.007	0.02
**Cu**	0.044	0.13
**Zn**	0.065	0.20
**As**	0.108	0.33
**Y**	0.0001	0.0002
**Mo**	0.002	0.01
**Cd**	0.0001	0.0002
**Sn**	0.018	0.05
**Ba**	0.004	0.01
**W**	0.001	0.002
**Hg**	0.001	0.005
**Pb**	0.005	0.01

Table S2. Spearman correlations of hair concentrations of trace elements (TEs) evaluated in the study. P-values are presented in parentheses, and significant correlations are bolded.

	B	Sc	V	Cr	Co	Ni	Cu	Zn	As	Y	Mo	Cd	Sn	Ba	W	Hg
**Sc**	**0.30 (0.04)**															
**V**	0.18 (0.23)	0.21 (0.16)														
**Cr**	0.001 (0.99)	0.06 (0.68)	0.18 (0.23)													
**Co**	0.20 (0.19)	0.27 (0.08)	**0.73 (<0.001)**	0.16 (0.28)												
**Ni**	0.28 (0.06)	0.22 (0.15)	**0.44 (0.003)**	0.03 (0.86)	**0.48 (0.001)**											
**Cu**	**0.41 (0.005)**	0.28 (0.06)	**0.48 (0.001)**	0.05 (0.74)	**0.45 (0.002)**	**0.61 (<0.001)**										
**Zn**	**0.55 (<0.001)**	0.04 (0.78)	0.11 (0.47)	-0.05 (0.75)	**0.30 (0.046)**	**0.31 (0.04)**	0.25 (0.10)									
**As**	**0.42 (0.004)**	0.18 (0.23)	0.03 (0.86)	0.01 (0.97)	0.08 (0.58)	**0.33 (0.030)**	0.01 (0.96)	**0.54 (<0.001)**								
**Y**	**0.30 (0.049)**	**0.46 (0.001)**	**0.50 (<0.001)**	-0.07 (0.66)	**0.67 (<0.001)**	**0.38 (0.010)**	**0.38 (0.010)**	**0.33 (0.030)**	0.20 (0.18)							
**Mo**	**0.53 (<0.001)**	0.26 (0.08)	**0.54 (<0.001)**	0.14 (0.37)	**0.35 (0.020)**	**0.42 (0.004)**	**0.56 (<0.001)**	0.25 (0.10)	0.15 (0.33)	**0.34 (0.02)**						
**Cd**	**0.34 (0.020)**	0.05 (0.75)	**0.49 (0.001)**	-0.13 (0.41)	**0.42 (0.004)**	**0.30 (0.046)**	**0.47 (0.001)**	0.15 (0.32)	-0.04 (0.78)	**0.30 (0.046)**	**0.50 (<0.001)**					
**Sn**	0.15 (0.33)	0.22 (0.15)	0.004 (0.98)	0.29 (0.06)	0.08 (0.62)	0.19 (0.22)	0.29 (0.05)	-0.08 (0.63)	0.21 (0.17)	0.11 (0.47)	0.22 (0.15)	-0.12 (0.45)				
**Ba**	0.28 (0.07)	0.19 (0.20)	0.26 (0.08)	0.08 (0.60)	**0.46 (0.001)**	**0.47 (0.001)**	0.13 (0.38)	**0.37 (0.010)**	**0.40 (0.006)**	**0.49 (0.001)**	0.15 (0.33)	-0.07 (0.65)	**0.39 (0.010)**			
**W**	0.19 (0.21)	0.09 (0.56)	0.17 (0.27)	0.11 (0.48)	0.01 (0.96)	0.11 (0.49)	0.08 (0.58)	0.04 (0.78)	0.19 (0.21)	-0.12 (0.44)	0.28 (0.06)	0.25 (0.10)	-0.07 (0.64)	-0.22 (0.15)		
**Hg**	0.18 (0.23)	0.14 (0.37)	-0.02 (0.92)	-0.13 (0.39)	0.10 (0.52)	0.16 (0.28)	-0.05 (0.74)	**0.43 (0.003)**	0.28 (0.06)	0.26 (0.09)	0.02 (0.90)	-0.13 (0.38)	-0.02 (0.88)	**0.48 (0.001)**	-0.18 (0.25)	
**Pb**	**0.54 (<0.001)**	0.16 (0.30)	**0.50 (0.001)**	0.07 (0.65)	**0.34 (0.020)**	0.08 (0.61)	**0.34 (0.020)**	0.16 (0.30)	0.12 (0.43)	0.27 (0.08)	**0.65 (<0.001)**	**0.66 (<0.001)**	0.09 (0.58)	-0.04 (0.78)	**0.45 (0.002)**	-0.10 (0.50)

Table S3. Associations between trace element concentrations and cell type proportion estimates. Significant associations (p-value < 0.05) are bolded.

**Trace element** (Log2)	White blood cell B-value (p-value)					
	**CD4+ T cells**	**CD8+ T cells**	**NK**	**B cells**	**Monocytes**	**Granulocytes**
**B**	-1.39 (0.736)	5.11 (0.247)	-3.57 (0.216)	-8.60 (0.276)	4.09 (0.539)	1.08 (0.608)
**Sc**	0.30 (0.913)	3.06 (0.289)	-2.49 (0.188)	0.48 (0.927)	1.66 (0.704)	0.15 (0.916)
**V**	-3.79 (0.399)	8.38 (0.078)	-0.74 (0.815)	2.52 (0.770)	9.56 (0.183)	-1.48 (0.520)
**Cr**	-2.64 (0.655)	-1.53 (0.810)	3.12 (0.454)	11.41 (0.314)	12.13 (0.200)	-2.43 (0.422)
**Co**	-2.68 (0.505)	2.63 (0.543)	-0.48 (0.867)	6.68 (0.387)	**17.64 (0.004)**	-1.79 (0.383)
**Ni**	-3.18 (0.497)	2.69 (0.593)	-1.51 (0.646)	-3.69 (0.682)	14.12 (0.056)	-0.24 (0.920)
**Cu**	-2.43 (0.538)	**8.31 (0.046)**	0.93 (0.739)	-4.62 (0.543)	9.70 (0.123)	-2.19 (0.277)
**Zn**	-4.98 (0.242)	2.98 (0.518)	-5.53 (0.062)	-11.62 (0.154)	11.93 (0.078)	3.04 (0.162)
**As**	-2.86 (0.743)	-7.15 (0.444)	-10.50 (0.082)	-21.69 (0.191)	-0.19 (0.989)	8.32 (0.057)
**Y**	-4.41 (0.311)	6.15 (0.187)	-1.63 (0.597)	-4.87 (0.561)	11.79 (0.089)	-0.17 (0.940)
**Mo**	-3.65 (0.345)	5.41 (0.192)	-1.55 (0.571)	-2.64 (0.724)	8.50 (0.170)	-0.16 (0.934)
**Cd**	-1.75 (0.670)	**9.41 (0.029)**	-2.00 (0.490)	-2.50 (0.752)	**12.89 (0.046)**	-1.74 (0.406)
**Sn**	0.40 (0.939)	5.00 (0.370)	4.36 (0.230)	-4.96 (0.619)	2.24 (0.789)	-3.36 (0.203)
**Ba**	-1.09 (0.856)	3.50 (0.588)	-3.41 (0.419)	-10.28 (0.372)	5.44 (0.574)	1.32 (0.667)
**W**	-1.65 (0.706)	0.22 (0.962)	2.19 (0.478)	-11.28 (0.176)	-3.14 (0.656)	0.20 (0.928)
**Hg**	3.95 (0.414)	4.54 (0.382)	-5.44 (0.106)	5.77 (0.535)	-5.10 (0.513)	0.88 (0.723)
**Pb**	-2.59 (0.659)	4.19 (0.501)	0.52 (0.901)	14.46 (0.195)	7.81 (0.406)	-0.46 (0.878)

NK: Natural killer. B-value: regression coefficient.

Figure S1. Q-Q plots obtained for the trace element evaluated in this study.

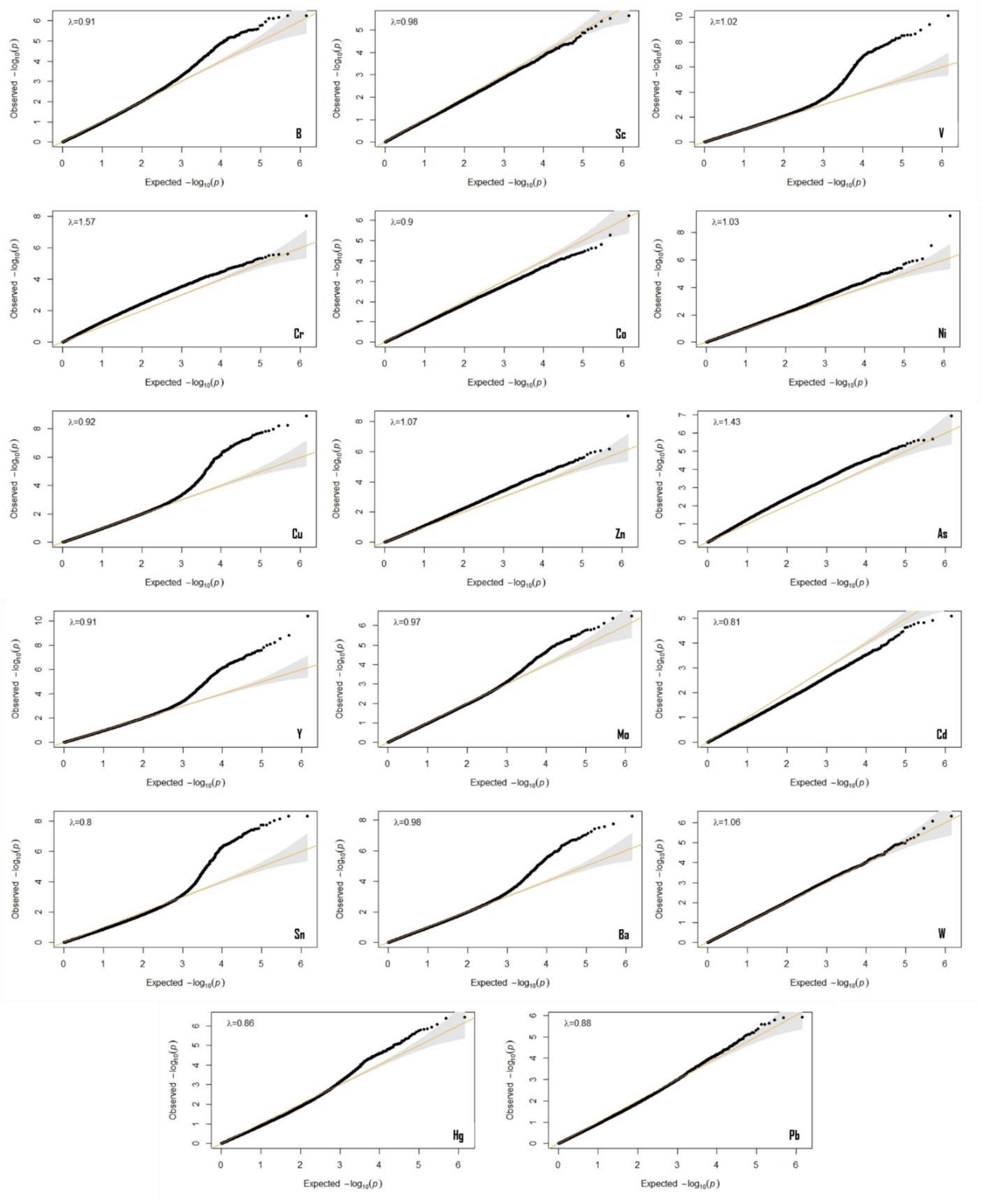

Table S4. DMPs associated with V (FDR < 0.05) adjusted for age, BMI, mother education, and estimated cell type proportions.

p-value	Chr	Pos	CpG	Log FC	FDR	Relation_to_Island	Gene
7.34E-11	7	128697416	cg06396390	0.41	0.000	S_Shore	
3.8E-10	1	50883329	cg22711792	0.49	0.000	Island	*DMRTA2*
1.02E-09	1	91192798	cg14663264	0.40	0.001	Island	
2.21E-09	20	36628932	cg01720774	0.37	0.002	OpenSea	*TTI1*
2.49E-09	5	93954434	cg11339965	0.41	0.002	Island	*ANKRD32*
2.53E-09	2	45870883	cg08715805	0.54	0.002	Island	
2.91E-09	1	161981053	cg27316795	0.69	0.002	OpenSea	*OLFML2B*
3.03E-09	17	3704574	cg13984928	-0.40	0.002	OpenSea	*ITGAE*
4.17E-09	10	17051623	cg20434599	0.55	0.003	OpenSea	*CUBN*
4.69E-09	8	81398656	cg22380165	0.51	0.003	Island	*ZBTB10*
4.83E-09	1	31474920	cg02771649	0.61	0.003	OpenSea	*PUM1*
6.63E-09	3	135969053	cg26010175	0.56	0.005	Island	*PCCB*
7.44E-09	17	46894684	cg12027420	0.44	0.005	Island	*TTLL6*
9.53E-09	10	99052468	cg06456389	0.75	0.007	Island	*ARHGAP19*
1.09E-08	13	97422029	cg09122588	0.84	0.008	OpenSea	*HS6ST3*
1.32E-08	16	87309686	cg05541311	0.49	0.009	OpenSea	
1.37E-08	1	241779250	cg03217228	0.64	0.010	OpenSea	*OPN3*
1.44E-08	1	158978812	cg04805131	-0.30	0.010	OpenSea	*IFI16*
1.63E-08	8	27779092	cg11133658	0.51	0.012	Island	*SCARA5*
1.66E-08	11	31530876	cg15088759	0.45	0.012	N_Shore	*ELP4*
1.72E-08	2	242120289	cg13734948	0.50	0.012	OpenSea	*PPP1R7*
1.92E-08	1	109582945	cg03677021	-0.42	0.014	N_Shore	*WDR47*
2.12E-08	11	115631394	cg25659317	0.52	0.015	S_Shore	*LINC00900*
2.12E-08	2	75708900	cg17706091	0.34	0.015	OpenSea	
2.32E-08	12	51419973	cg04118903	-0.29	0.017	Island	*SLC11A2*
2.37E-08	1	41960295	cg09548495	0.56	0.017	N_Shore	
2.6E-08	1	224803364	cg09558034	0.53	0.019	N_Shore	*CNIH3*
2.85E-08	1	1957711	cg10837110	-0.48	0.020	N_Shore	*GABRD*
2.98E-08	5	39373418	cg02653030	0.38	0.021	OpenSea	*DAB2*
2.98E-08	8	1840794	cg24613789	0.39	0.021	OpenSea	*ARHGEF10*
3.03E-08	16	19890592	cg01620602	0.40	0.022	OpenSea	*GPRC5B*
3.22E-08	5	56246733	cg08288226	0.29	0.023	Island	*MIER3*
3.28E-08	8	28535162	cg12221428	0.44	0.024	OpenSea	
3.33E-08	13	90045847	cg24712244	-0.24	0.024	OpenSea	
3.87E-08	5	122947164	cg03722513	-0.23	0.028	OpenSea	*CSNK1G3*
3.99E-08	15	65068371	cg12725420	0.56	0.029	S_Shore	*RBPMS2*
4.14E-08	2	233285289	cg13193840	0.51	0.030	Island	
4.14E-08	19	30862847	cg15977798	-0.21	0.030	N_Shelf	*ZNF536*

p-value	Chr	Pos	CpG	Log FC	FDR	Relation_to_Island	Gene
4.92E-08	17	78822725	cg13945540	0.43	0.035	OpenSea	*RPTOR*
5.46E-08	3	138830878	cg14638147	0.38	0.039	OpenSea	*BPESC1*
5.46E-08	19	18946913	cg15482025	0.40	0.039	S_Shelf	*UPF1*
5.5E-08	3	78079111	cg10728960	0.50	0.039	OpenSea	
5.62E-08	18	47123950	cg12490577	0.39	0.040	OpenSea	
5.81E-08	17	2652918	cg09652807	-0.46	0.042	Island	*MIR1253*
5.86E-08	16	2529732	cg26531633	0.28	0.042	S_Shelf	*TBC1D24*
5.92E-08	12	113440283	cg24531077	0.42	0.043	OpenSea	*OAS2*
5.97E-08	6	10690667	cg07606551	0.24	0.043	N_Shelf	*C6orf52*
6.14E-08	8	1330464	cg03215535	0.65	0.044	S_Shelf	
6.17E-08	8	144775130	cg05342816	0.38	0.044	N_Shore	*ZNF707*
6.21E-08	18	6543922	cg01786044	0.38	0.045	OpenSea	*LINC01387*
6.53E-08	20	54987140	cg24587601	0.31	0.047	OpenSea	*CASS4*
6.58E-08	2	38831686	cg02565468	0.38	0.047	S_Shore	
6.59E-08	3	72701850	cg07127883	0.39	0.047	N_Shelf	
6.82E-08	1	155829128	cg11545364	0.42	0.049	Island	*SYT11*
6.86E-08	16	56485455	cg14837652	0.34	0.049	Island	*NUDT21*
7.5E-08	6	132722778	cg13640626	0.46	0.054	Island	*MOXD1*
8.63E-08	5	94891623	cg03977394	0.46	0.062	S_Shore	*ARSK*
9.08E-08	6	589274	cg14653814	0.19	0.065	OpenSea	*EXOC2*
9.12E-08	9	77461194	cg05614767	-0.26	0.065	OpenSea	*TRPM6*
9.49E-08	3	196050645	cg27088176	0.59	0.068	OpenSea	*TM4SF19*
9.59E-08	5	172566090	cg25152909	0.36	0.069	OpenSea	*C5orf41*
9.65E-08	12	124121050	cg12017810	0.30	0.069	S_Shelf	*GTF2H3*
1.03E-07	1	227585342	cg12245524	0.36	0.074	OpenSea	
1.08E-07	9	80003456	cg26251415	0.49	0.078	OpenSea	*VPS13A*
1.1E-07	18	19799634	cg15651884	0.42	0.079	OpenSea	
1.11E-07	17	25619946	cg07564598	0.42	0.079	N_Shore	*WSB1*
1.14E-07	22	38541473	cg19871462	0.43	0.082	OpenSea	*PLA2G6*
1.24E-07	6	166205748	cg13220290	0.30	0.089	OpenSea	
1.26E-07	5	73463484	cg01195564	0.25	0.090	OpenSea	
1.27E-07	2	137950714	cg17180863	1.04	0.091	OpenSea	*THSD7B*
1.44E-07	3	47563649	cg04507495	-0.42	0.104	OpenSea	
1.53E-07	17	599829	cg12281099	0.38	0.110	OpenSea	*VPS53*
1.67E-07	6	29442830	cg10619365	0.37	0.120	OpenSea	
1.71E-07	14	58619219	cg18463820	0.46	0.123	Island	*C14orf37*
1.77E-07	3	81792982	cg02639218	0.28	0.127	OpenSea	*GBE1*
1.84E-07	14	104400143	cg24270471	0.29	0.132	OpenSea	*TDRD9*
1.87E-07	18	35101941	cg18032297	0.35	0.134	N_Shelf	*BRUNOL4*
1.87E-07	9	96272718	cg00688269	0.27	0.134	OpenSea	*FAM120A*

p-value	Chr	Pos	CpG	Log FC	FDR	Relation_to_Island	Gene
2.02E-07	4	186541367	cg04764474	0.52	0.145	N_Shelf	*SORBS2*
2.03E-07	17	49450892	cg02960148	0.33	0.146	OpenSea	
2.15E-07	16	67380005	cg03956914	-0.23	0.154	OpenSea	*LRRC36*
2.24E-07	1	153643632	cg13209085	0.45	0.161	S_Shore	*ILF2*
2.36E-07	5	114763264	cg17570719	0.39	0.170	OpenSea	
2.52E-07	19	35606534	cg01408817	-0.12	0.181	OpenSea	*FXYD3*
2.53E-07	14	76070515	cg10993988	-0.23	0.182	OpenSea	*MIR7641-2*
2.53E-07	9	133706938	cg14483856	-0.15	0.182	N_Shelf	*ABL1*
2.72E-07	11	134681766	cg16729283	0.36	0.195	OpenSea	
2.8E-07	9	136739643	cg09136786	0.33	0.201	OpenSea	*VAV2*
2.82E-07	7	27155002	cg16748008	0.34	0.202	Island	*HOXA3*
2.95E-07	21	43324038	cg02590715	0.37	0.212	N_Shelf	*C2CD2*
3.08E-07	10	47667303	cg26231261	0.33	0.221	OpenSea	*ANTXRL*
3.1E-07	17	8815841	cg24251850	0.25	0.222	OpenSea	*PIK3R5*
3.11E-07	7	128415071	cg09658621	0.38	0.223	OpenSea	*OPN1SW*
3.29E-07	2	150571527	cg22274628	-0.21	0.236	OpenSea	*LOC101929231*
3.39E-07	3	14200360	cg08054515	0.26	0.243	OpenSea	*XPC*
3.5E-07	7	99772832	cg13246281	0.28	0.251	N_Shore	*GPC2*
3.65E-07	11	95779454	cg25267808	0.39	0.262	OpenSea	*MAML2*
3.71E-07	7	27155039	cg18680977	0.41	0.266	Island	*HOXA3*
4.49E-07	10	180329	cg02867818	0.39	0.322	N_Shore	*ZMYND11*
4.84E-07	10	72647938	cg25757740	0.41	0.348	Island	*PCBD1*
4.95E-07	19	3866347	cg19705859	0.30	0.356	N_Shelf	*ZFR2*
5.21E-07	17	66286791	cg07407787	0.14	0.374	Island	*ARSG*
5.54E-07	8	110342178	cg27574309	-0.25	0.398	N_Shelf	*NUDCD1*
5.83E-07	9	138145731	cg13818014	-0.59	0.419	OpenSea	
6.32E-07	11	67829755	cg06724394	0.29	0.454	OpenSea	*CHKA*
6.38E-07	18	44373583	cg08113537	0.34	0.458	OpenSea	
6.47E-07	17	81018426	cg08040148	-0.43	0.465	S_Shelf	
6.75E-07	15	76151117	cg11384517	0.40	0.484	OpenSea	*UBE2Q2*
6.8E-07	13	111907965	cg07073749	-0.23	0.488	S_Shore	*ARHGEF7*
7.19E-07	7	27155036	cg16406967	0.38	0.516	Island	*HOXA3*
7.21E-07	12	133198653	cg21162817	0.38	0.518	S_Shelf	*P2RX2*
7.22E-07	10	12742170	cg01167366	0.81	0.519	OpenSea	*CAMK1D*
7.3E-07	20	18277898	cg18896550	-0.21	0.524	OpenSea	*ZNF133*
7.37E-07	14	22217354	cg00208384	0.48	0.529	OpenSea	
7.51E-07	7	130138018	cg14923015	-0.14	0.539	OpenSea	*MEST*
9.41E-07	11	94565946	cg01382930	-0.23	0.676	OpenSea	*AMOTL1*
9.47E-07	22	39899355	cg22773662	0.51	0.680	S_Shore	*MIEF1*
9.76E-07	6	33281712	cg23381664	0.33	0.700	Island	*TAPBP*

p-value	Chr	Pos	CpG	Log FC	FDR	Relation_to_Island	Gene
1.05E-06	14	90399131	cg09993641	-0.23	0.751	OpenSea	*EFCAB11*
1.05E-06	14	102176671	cg19730972	-0.39	0.757	S_Shelf	
1.11E-06	19	49994372	cg09372739	0.35	0.797	S_Shelf	*SNORD35A*
1.12E-06	8	40059435	cg20660197	0.30	0.805	OpenSea	
1.16E-06	2	52524614	cg00633008	0.25	0.834	OpenSea	
1.22E-06	1	53207187	cg09509462	-0.30	0.875	OpenSea	*ZYG11B*
1.3E-06	2	18705868	cg04497514	0.32	0.930	OpenSea	
1.45E-06	6	108977419	cg09546902	0.36	1.000	OpenSea	*FOXO3*
1.45E-06	14	70242852	cg14059534	0.34	1.000	OpenSea	*SLC10A1*
1.46E-06	14	92927875	cg11107966	-0.34	1.000	OpenSea	*SLC24A4*
1.52E-06	10	104436518	cg27316828	0.52	1.000	OpenSea	*ARL3*
1.53E-06	15	101641336	cg06375969	0.31	1.000	OpenSea	
1.58E-06	5	118332221	cg17136442	0.39	1.000	OpenSea	
1.6E-06	12	98811404	cg23053746	0.27	1.000	OpenSea	
1.66E-06	1	114212812	cg04141901	0.26	1.000	OpenSea	*MAGI3*
1.83E-06	10	111967167	cg21196958	0.42	1.000	N_Shore	*MXI1*
1.87E-06	10	135049999	cg04652645	0.40	1.000	Island	*VENTX*
1.91E-06	13	95846842	cg00241432	0.35	1.000	OpenSea	*ABCC4*
1.92E-06	14	107078376	cg00715313	-0.40	1.000	OpenSea	
1.94E-06	16	89162377	cg06359492	0.29	1.000	S_Shore	*ACSF3*
2.05E-06	4	78774513	cg16526120	0.48	1.000	OpenSea	
2.06E-06	8	103819572	cg03547517	0.70	1.000	S_Shore	
2.09E-06	8	75288417	cg00190393	-0.25	1.000	OpenSea	
2.2E-06	13	74512388	cg18662671	0.27	1.000	OpenSea	*KLF12*
2.33E-06	2	179315654	cg17833257	0.34	1.000	Island	*DFNB59*
2.41E-06	7	27154914	cg00318947	0.28	1.000	N_Shore	*HOXA3*
2.47E-06	2	39512317	cg21990552	-0.24	1.000	OpenSea	*MAP4K3*
2.52E-06	12	104378097	cg27454347	-0.20	1.000	OpenSea	*TDG*
2.69E-06	5	110848437	cg11890437	0.21	1.000	Island	*STARD4*
2.81E-06	4	100003288	cg23372626	0.26	1.000	OpenSea	*ADH5*
2.97E-06	3	182561316	cg15187789	-0.20	1.000	OpenSea	*ATP11B*
3.01E-06	3	182591616	cg05002500	-0.16	1.000	OpenSea	*ATP11B*
3.02E-06	12	125534043	cg13938881	0.52	1.000	N_Shore	
3.06E-06	9	35828874	cg01543404	0.34	1.000	Island	*TMEM8B*
3.09E-06	3	183995462	cg07131336	0.27	1.000	S_Shore	*ECE2*
3.12E-06	19	16629806	cg14798382	-0.40	1.000	N_Shore	*CHERP*
3.29E-06	18	46621257	cg20465758	-0.16	1.000	OpenSea	*DYM*
3.29E-06	4	148401273	cg16264526	-0.19	1.000	N_Shore	*EDNRA*
3.32E-06	7	27154911	cg15982700	0.33	1.000	N_Shore	*HOXA3*
3.36E-06	3	44912362	cg02449689	0.30	1.000	OpenSea	

p-value	Chr	Pos	CpG	Log FC	FDR	Relation_to_Island	Gene
3.36E-06	16	103646	cg09969830	-0.19	1.000	Island	*SNRNP25*
3.48E-06	1	53556701	cg08607780	-0.35	1.000	N_Shore	*SLC1A7*
3.84E-06	6	150329845	cg27001715	-0.73	1.000	S_Shelf	
3.84E-06	12	122669186	cg08120831	0.12	1.000	S_Shore	*LRRC43*
3.94E-06	11	3735574	cg09537015	0.22	1.000	OpenSea	*NUP98*
4.17E-06	1	40946609	cg00557440	0.22	1.000	S_Shelf	*ZNF642*
4.26E-06	19	51525638	cg09923953	-0.16	1.000	S_Shelf	*KLK11*
4.34E-06	4	3533876	cg16565635	-0.33	1.000	Island	*LRPAP1*
4.34E-06	5	150936730	cg06071063	0.27	1.000	OpenSea	*FAT2*
4.7E-06	1	234211980	cg08885541	0.30	1.000	OpenSea	*SLC35F3*
4.81E-06	3	178055626	cg13609889	-0.21	1.000	OpenSea	
4.93E-06	1	112533012	cg21348554	0.20	1.000	Island	*KCND3*
4.98E-06	6	39899321	cg02680909	-0.13	1.000	N_Shelf	*MOCS1*
5.19E-06	10	112466738	cg13331591	0.28	1.000	OpenSea	*RBM20*
5.48E-06	5	157702593	cg18702684	0.23	1.000	OpenSea	
5.56E-06	1	203456818	cg03336727	0.44	1.000	Island	*PRELP*
5.69E-06	12	92836720	cg05914217	0.29	1.000	OpenSea	
5.94E-06	16	85317070	cg27079934	0.31	1.000	N_Shore	*LINC00311*
6.02E-06	14	74680453	cg12779982	0.39	1.000	N_Shelf	
6.04E-06	1	92764777	cg24501027	0.14	1.000	Island	*RPAP2*
6.04E-06	17	57228640	cg02611741	-0.20	1.000	N_Shelf	*MIR301A*
6.06E-06	11	1945648	cg11654118	0.31	1.000	N_Shore	*TNNT3*
6.09E-06	8	6603212	cg06460618	0.30	1.000	OpenSea	*MIR4659B*
6.11E-06	2	109204687	cg02385799	0.38	1.000	OpenSea	*LIMS1*
6.3E-06	12	92384227	cg08921760	-0.23	1.000	OpenSea	*C12orf79*
6.41E-06	3	101877174	cg22728989	-0.18	1.000	OpenSea	
6.83E-06	9	37780478	cg13441107	0.18	1.000	OpenSea	*EXOSC3*
6.85E-06	10	75012760	cg03037366	0.30	1.000	S_Shore	*MRPS16*
7.25E-06	9	34701531	cg14583869	-0.17	1.000	Island	
7.73E-06	1	234509059	cg24557133	0.36	1.000	Island	*C1orf31*
7.76E-06	18	75847666	cg17951166	1.12	1.000	OpenSea	
7.77E-06	16	85646973	cg01530687	0.13	1.000	Island	*KIAA0182*
8.27E-06	6	35887678	cg00087417	0.23	1.000	N_Shore	*SRPK1*
8.33E-06	1	11405255	cg03007239	-0.09	1.000	OpenSea	
8.6E-06	9	95055763	cg25358410	0.29	1.000	Island	*IARS*
8.92E-06	15	63116127	cg18065177	-0.15	1.000	OpenSea	*TLN2*
8.93E-06	2	73964144	cg07852972	0.27	1.000	N_Shore	*TPRKB*
9.01E-06	1	65525290	cg14871564	0.22	1.000	OpenSea	*MIR101-1*
9.06E-06	2	65356625	cg24614854	0.12	1.000	N_Shore	*RAB1A*
9.08E-06	17	75309501	cg16785556	-0.12	1.000	OpenSea	*sep-09*

p-value	Chr	Pos	CpG	Log FC	FDR	Relation_to_Island	Gene
9.25E-06	6	146214507	cg08433435	-0.27	1.000	OpenSea	*SHPRH*
9.27E-06	7	27154845	cg16644023	0.32	1.000	N_Shore	*HOXA3*
9.46E-06	7	45961849	cg05083496	0.43	1.000	S_Shore	*IGFBP3*
9.65E-06	5	149526679	cg05526146	-0.12	1.000	OpenSea	*PDGFRB*
9.76E-06	3	149673434	cg03645730	-0.37	1.000	OpenSea	*RNF13*
9.85E-06	19	44278274	cg03731131	0.18	1.000	Island	*KCNN4*
1.01E-05	1	119933714	cg09651697	0.26	1.000	OpenSea	*HAO2*
1.02E-05	16	732232	cg17546868	0.27	1.000	Island	*STUB1*
1.03E-05	14	88200397	cg18174099	0.29	1.000	OpenSea	
1.03E-05	2	228523749	cg06526280	-0.15	1.000	OpenSea	
1.04E-05	2	201327726	cg09723782	-0.20	1.000	OpenSea	*SPATS2L*
1.11E-05	19	6268276	cg08520648	0.30	1.000	N_Shelf	*MLLT1*
1.11E-05	2	1232428	cg16653700	-0.24	1.000	OpenSea	*SNTG2*
1.13E-05	10	134803138	cg18855429	-0.15	1.000	N_Shore	
1.2E-05	4	148671546	cg11745567	0.33	1.000	OpenSea	*ARHGAP10*
1.21E-05	19	1822445	cg17380091	0.16	1.000	Island	*REXO1*
1.21E-05	1	148596792	cg06441514	-0.18	1.000	N_Shore	
1.22E-05	2	201981068	cg14173083	0.11	1.000	Island	*CFLAR*
1.28E-05	3	189727313	cg05016848	-0.20	1.000	OpenSea	*P3H2*
1.33E-05	2	71738112	cg04819611	0.66	1.000	OpenSea	*DYSF*
1.33E-05	20	20202259	cg22119133	-0.16	1.000	OpenSea	*CFAP61*
1.36E-05	16	53164514	cg06288634	0.36	1.000	Island	*CHD9*
1.43E-05	6	66804830	cg22034507	-0.26	1.000	Island	
1.51E-05	11	66682555	cg20966316	0.15	1.000	OpenSea	*PC*
1.52E-05	2	231068094	cg05408985	0.29	1.000	OpenSea	*SP110*
1.52E-05	19	36432051	cg04918389	0.18	1.000	S_Shore	*LRFN3*
1.57E-05	6	97638598	cg15778867	-0.25	1.000	OpenSea	*MIR548H3*

Chr: Chromosome. Pos: Position. Log FC: Log fold change.

Table S5. DMPs associated with Cu (FDR < 0.05) adjusted for age, BMI, mother education, and estimated cell type proportions.

***p*-value**	**Chr**	**Pos**	**CpG**	**Log FC**	**FDR**	**Relation_to_Island**	**Gene**
1.21E-09	12	113440283	cg24531077	0.54	0.001	OpenSea	*OAS2*
5.79E-09	3	47563649	cg04507495	-0.54	0.002	OpenSea	
6.37E-09	17	3704574	cg13984928	-0.47	0.002	OpenSea	*ITGAE*
1.08E-08	5	172566090	cg25152909	0.45	0.002	OpenSea	*C5orf41*
1.32E-08	7	27154914	cg00318947	0.38	0.002	N_Shore	*HOXA3*
1.52E-08	8	144775130	cg05342816	0.47	0.002	N_Shore	*ZNF707*
1.75E-08	10	17051623	cg20434599	0.64	0.002	OpenSea	*CUBN*
2.03E-08	6	29442830	cg10619365	0.47	0.002	OpenSea	
2.23E-08	13	97422029	cg09122588	0.99	0.002	OpenSea	*HS6ST3*
2.29E-08	12	51419973	cg04118903	-0.34	0.002	Island	*SLC11A2*
2.76E-08	1	41960295	cg09548495	0.67	0.002	N_Shore	
2.91E-08	7	128415071	cg09658621	0.48	0.002	OpenSea	*OPN1SW*
3.87E-08	4	3533876	cg16565635	-0.45	0.002	Island	*LRPAP1*
4.25E-08	18	19799634	cg15651884	0.52	0.002	OpenSea	
5.09E-08	4	186541367	cg04764474	0.65	0.002	N_Shelf	*SORBS2*
5.24E-08	17	49450892	cg02960148	0.41	0.002	OpenSea	
5.53E-08	9	138145731	cg13818014	-0.74	0.002	OpenSea	
5.95E-08	2	109204687	cg02385799	0.51	0.002	OpenSea	*LIMS1*
6.06E-08	9	136739643	cg09136786	0.41	0.002	OpenSea	*VAV2*
6.19E-08	5	39373418	cg02653030	0.45	0.002	OpenSea	*DAB2*
6.42E-08	10	104436518	cg27316828	0.68	0.002	OpenSea	*ARL3*
7.56E-08	22	38541473	cg19871462	0.52	0.002	OpenSea	*PLA2G6*
7.85E-08	10	12742170	cg01167366	1.02	0.002	OpenSea	*CAMK1D*
8.5E-08	11	3735574	cg09537015	0.29	0.002	OpenSea	*NUP98*
8.6E-08	1	31474920	cg02771649	0.69	0.002	OpenSea	*PUM1*
8.75E-08	2	242120289	cg13734948	0.58	0.002	OpenSea	*PPP1R7*
9.48E-08	12	133198653	cg21162817	0.47	0.003	S_Shelf	*P2RX2*
1.03E-07	3	196050645	cg27088176	0.71	0.003	OpenSea	*TM4SF19*
1.09E-07	5	114763264	cg17570719	0.47	0.003	OpenSea	
1.16E-07	2	38831686	cg02565468	0.45	0.003	S_Shore	
1.19E-07	22	39899355	cg22773662	0.64	0.003	S_Shore	*MIEF1*
1.23E-07	16	732232	cg17546868	0.36	0.003	Island	*STUB1*
1.29E-07	11	134681766	cg16729283	0.43	0.003	OpenSea	
1.45E-07	4	3533922	cg23528723	-0.40	0.003	Island	*LRPAP1*
1.49E-07	2	201981068	cg14173083	0.14	0.003	Island	*CFLAR*
1.57E-07	19	18946913	cg15482025	0.47	0.003	S_Shelf	*UPF1*
1.59E-07	10	47667303	cg26231261	0.40	0.003	OpenSea	*ANTXRL*
1.64E-07	5	150936730	cg06071063	0.35	0.003	OpenSea	*FAT2*
1.66E-07	17	78822725	cg13945540	0.50	0.003	OpenSea	*RPTOR*

p-value	Chr	Pos	CpG	Log FC	FDR	Relation_to_Island	Gene
1.68E-07	8	1330464	cg03215535	0.76	0.003	S_Shelf	
1.76E-07	5	93954434	cg11339965	0.45	0.003	Island	*ANKRD32*
1.92E-07	7	128697416	cg06396390	0.43	0.003	S_Shore	
2.11E-07	1	224803364	cg09558034	0.61	0.004	N_Shore	*CNIH3*
2.18E-07	10	99052468	cg06456389	0.85	0.004	Island	*ARHGAP19*
2.3E-07	12	63479872	cg04774139	-0.26	0.004	OpenSea	
2.46E-07	6	166205748	cg13220290	0.36	0.004	OpenSea	
2.63E-07	13	95846842	cg00241432	0.44	0.004	OpenSea	*ABCC4*
2.66E-07	1	91192798	cg14663264	0.43	0.004	Island	
2.75E-07	17	14642137	cg05801153	0.44	0.004	OpenSea	
2.8E-07	1	155036577	cg23008177	0.37	0.004	Island	*EFNA4*
2.86E-07	1	241779250	cg03217228	0.72	0.004	OpenSea	*OPN3*
3.02E-07	1	109582945	cg03677021	-0.47	0.004	N_Shore	*WDR47*
3.27E-07	15	65068371	cg12725420	0.64	0.004	S_Shore	*RBPMS2*
3.27E-07	7	27155036	cg16406967	0.46	0.004	Island	*HOXA3*
3.32E-07	7	27155039	cg18680977	0.49	0.004	Island	*HOXA3*
3.36E-07	20	56135029	cg10278454	0.34	0.004	OpenSea	*PCK1*
3.38E-07	7	27155002	cg16748008	0.40	0.004	Island	*HOXA3*
3.45E-07	18	46621257	cg20465758	-0.20	0.004	OpenSea	*DYM*
3.5E-07	21	43324038	cg02590715	0.45	0.004	N_Shelf	*C2CD2*
3.51E-07	3	78079111	cg10728960	0.57	0.004	OpenSea	
3.93E-07	16	19890592	cg01620602	0.45	0.005	OpenSea	*GPRC5B*
3.97E-07	11	65211709	cg13167340	-0.41	0.005	OpenSea	*MIR612*
3.98E-07	14	59760772	cg11747104	-0.23	0.005	OpenSea	*DAAM1*
4.07E-07	2	45870883	cg08715805	0.58	0.005	Island	
4.11E-07	5	73463484	cg01195564	0.29	0.005	OpenSea	
4.18E-07	2	75708900	cg17706091	0.38	0.005	OpenSea	
4.39E-07	5	14038630	cg10979364	-0.17	0.005	OpenSea	
4.79E-07	12	76159482	cg24835883	0.39	0.005	OpenSea	
4.94E-07	2	113341685	cg11317677	0.39	0.005	Island	*CHCHD5*
5.84E-07	12	124121050	cg12017810	0.34	0.006	S_Shelf	*GTF2H3*
6.04E-07	1	161981053	cg27316795	0.74	0.006	OpenSea	*OLFML2B*
6.77E-07	17	2652918	cg09652807	-0.52	0.007	Island	*MIR1253*
6.84E-07	1	234211980	cg08885541	0.39	0.007	OpenSea	*SLC35F3*
6.99E-07	8	6603212	cg06460618	0.39	0.007	OpenSea	*MIR4659B*
7.08E-07	3	170584674	cg16620263	-0.56	0.007	N_Shelf	*RPL22L1*
7.14E-07	13	90045847	cg24712244	-0.27	0.007	OpenSea	
7.37E-07	6	10690667	cg07606551	0.28	0.007	N_Shelf	*C6orf52*
7.66E-07	12	98811404	cg23053746	0.33	0.007	OpenSea	
8.03E-07	6	31647562	cg21561281	0.39	0.007	N_Shore	*LY6G5C*

p-value	Chr	Pos	CpG	Log FC	FDR	Relation_to_Island	Gene
8.2E-07	2	233285289	cg13193840	0.57	0.007	Island	
8.36E-07	16	103646	cg09969830	-0.24	0.007	Island	*SNRNP25*
8.81E-07	14	90399131	cg09993641	-0.28	0.008	OpenSea	*EFCAB11*
9.03E-07	10	72647938	cg25757740	0.48	0.008	Island	*PCBD1*
9.05E-07	19	49994372	cg09372739	0.43	0.008	S_Shelf	*SNORD35A*
9.62E-07	11	115631394	cg25659317	0.57	0.008	S_Shore	*LINC00900*
9.79E-07	19	30862847	cg15977798	-0.24	0.008	N_Shelf	*ZNF536*
1.01E-06	11	31530876	cg15088759	0.49	0.008	N_Shore	*ELP4*
1.01E-06	8	75288417	cg00190393	-0.31	0.008	OpenSea	
1.02E-06	11	67829755	cg06724394	0.34	0.008	OpenSea	*CHKA*
1.03E-06	2	102026463	cg07099118	-0.31	0.008	OpenSea	*RFX8*
1.04E-06	17	8815841	cg24251850	0.29	0.008	OpenSea	*PIK3R5*
1.08E-06	11	1945648	cg11654118	0.39	0.008	N_Shore	*TNNT3*
1.11E-06	8	28535162	cg12221428	0.49	0.009	OpenSea	
1.14E-06	18	47123950	cg12490577	0.43	0.009	OpenSea	
1.17E-06	1	114212812	cg04141901	0.32	0.009	OpenSea	*MAGI3*
1.19E-06	16	56485455	cg14837652	0.38	0.009	Island	*NUDT21*
1.22E-06	15	76151117	cg11384517	0.47	0.009	OpenSea	*UBE2Q2*
1.22E-06	2	201327726	cg09723782	-0.25	0.009	OpenSea	*SPATS2L*
1.24E-06	13	111907965	cg07073749	-0.27	0.009	S_Shore	*ARHGEF7*
1.25E-06	19	6268276	cg08520648	0.39	0.009	N_Shelf	*MLLT1*
1.25E-06	2	113341711	cg26009035	0.30	0.009	Island	*CHCHD5*
1.26E-06	16	89162377	cg06359492	0.35	0.009	S_Shore	*ACSF3*
1.27E-06	7	27155173	cg04351734	0.40	0.009	Island	*HOXA3*
1.32E-06	18	44373583	cg08113537	0.40	0.009	OpenSea	
1.32E-06	9	80003456	cg26251415	0.55	0.009	OpenSea	*VPS13A*
1.35E-06	18	6543922	cg01786044	0.42	0.009	OpenSea	*LINC01387*
1.37E-06	6	108930710	cg26165197	0.28	0.009	OpenSea	*FOXO3*
1.38E-06	1	65619463	cg17537030	0.33	0.009	OpenSea	*AK4*
1.41E-06	17	81018426	cg08040148	-0.50	0.009	S_Shelf	
1.43E-06	3	44912362	cg02449689	0.36	0.009	OpenSea	
1.8E-06	5	118332221	cg17136442	0.47	0.012	OpenSea	
1.81E-06	2	170397488	cg13850024	-0.29	0.012	OpenSea	*FASTKD1*
1.98E-06	3	14200360	cg08054515	0.30	0.013	OpenSea	*XPC*
2.06E-06	16	87309686	cg05541311	0.52	0.013	OpenSea	
2.11E-06	10	180329	cg02867818	0.45	0.013	N_Shore	*ZMYND11*
2.11E-06	1	155829128	cg11545364	0.47	0.013	Island	*SYT11*
2.16E-06	19	3866347	cg19705859	0.34	0.013	N_Shelf	*ZFR2*
2.35E-06	5	21689217	cg26630199	-0.19	0.014	OpenSea	
2.38E-06	9	96272718	cg00688269	0.31	0.014	OpenSea	*FAM120A*

p-value	Chr	Pos	CpG	Log FC	FDR	Relation_to_Island	Gene
2.42E-06	4	55338905	cg21813288	0.27	0.014	OpenSea	
2.49E-06	1	50883329	cg22711792	0.49	0.015	Island	*DMRTA2*
2.52E-06	2	55792613	cg10358597	-0.22	0.015	OpenSea	*PPP4R3B*
2.56E-06	18	75847666	cg17951166	1.39	0.015	OpenSea	
2.56E-06	7	99772832	cg13246281	0.32	0.015	N_Shore	*GPC2*
2.68E-06	4	78774513	cg16526120	0.57	0.015	OpenSea	
2.7E-06	2	18705868	cg04497514	0.38	0.015	OpenSea	
2.71E-06	9	37780478	cg13441107	0.23	0.015	OpenSea	*EXOSC3*
2.91E-06	1	227585342	cg12245524	0.40	0.016	OpenSea	
3.19E-06	20	18277898	cg18896550	-0.24	0.018	OpenSea	*ZNF133*
3.23E-06	8	24240597	cg14143055	-0.22	0.018	OpenSea	*ADAMDEC1*
3.41E-06	1	41923683	cg02327542	0.15	0.019	OpenSea	
3.45E-06	17	599829	cg12281099	0.42	0.019	OpenSea	*VPS53*
3.8E-06	6	589274	cg14653814	0.20	0.020	OpenSea	*EXOC2*
3.93E-06	18	69503710	cg22773954	-0.33	0.021	OpenSea	
4.05E-06	8	38872513	cg04026002	-0.33	0.021	OpenSea	*ADAM9*
4.16E-06	3	81792982	cg02639218	0.31	0.022	OpenSea	*GBE1*
4.29E-06	7	27154845	cg16644023	0.39	0.022	N_Shore	*HOXA3*
4.38E-06	14	58619219	cg18463820	0.51	0.022	Island	*C14orf37*
4.39E-06	1	192597225	cg24700374	-0.29	0.022	OpenSea	
4.45E-06	8	27779092	cg11133658	0.53	0.023	Island	*SCARA5*
4.89E-06	3	72701850	cg07127883	0.42	0.025	N_Shelf	
4.97E-06	1	4107738	cg05079161	0.18	0.025	OpenSea	
5.11E-06	14	76070515	cg10993988	-0.25	0.025	OpenSea	*MIR7641-2*
5.38E-06	1	158174399	cg07377195	-0.40	0.026	OpenSea	
5.38E-06	17	25619946	cg07564598	0.45	0.026	N_Shore	*WSB1*
5.56E-06	11	259533	cg27354893	0.33	0.027	OpenSea	
5.57E-06	2	52524614	cg00633008	0.29	0.027	OpenSea	
5.71E-06	6	132722778	cg13640626	0.49	0.027	Island	*MOXD1*
5.87E-06	3	135969053	cg26010175	0.57	0.028	Island	*PCCB*
5.95E-06	5	94891623	cg03977394	0.50	0.028	S_Shore	*ARSK*
5.95E-06	5	157702593	cg18702684	0.27	0.028	OpenSea	
6.07E-06	16	2529732	cg26531633	0.30	0.028	S_Shelf	*TBC1D24*
6.17E-06	3	178055626	cg13609889	-0.25	0.029	OpenSea	
6.36E-06	1	53207187	cg09509462	-0.34	0.029	OpenSea	*ZYG11B*
6.41E-06	6	108977419	cg09546902	0.41	0.029	OpenSea	*FOXO3*
6.45E-06	18	35101941	cg18032297	0.38	0.029	N_Shelf	*BRUNOL4*
6.49E-06	2	73964144	cg07852972	0.33	0.029	N_Shore	*TPRKB*
6.56E-06	2	150571527	cg22274628	-0.23	0.029	OpenSea	*LOC101929 231*
6.57E-06	7	98642053	cg22045105	-0.26	0.029	OpenSea	*SMURF1*

p-value	Chr	Pos	CpG	Log FC	FDR	Relation_to_Island	Gene
6.73E-06	14	92927875	cg11107966	-0.39	0.030	OpenSea	*SLC24A4*
6.84E-06	1	1957711	cg10837110	-0.50	0.030	N_Shore	*GABRD*
6.89E-06	3	120214753	cg18324806	-0.19	0.030	OpenSea	
7.31E-06	8	81398656	cg22380165	0.51	0.032	Island	*ZBTB10*
7.31E-06	6	76782727	cg14836984	-0.21	0.032	OpenSea	*IMPG1*
7.43E-06	4	187621790	cg03301671	0.44	0.032	OpenSea	*FAT1*
7.52E-06	11	95779454	cg25267808	0.42	0.032	OpenSea	*MAML2*
7.76E-06	6	10434425	cg10264081	-0.14	0.033	OpenSea	*LINC00518*
7.82E-06	2	32581129	cg17615530	-0.20	0.033	N_Shore	*BIRC6*
7.94E-06	8	81787111	cg24636969	-0.56	0.033	S_Shore	*ZNF704*
8.37E-06	12	92384227	cg08921760	-0.27	0.035	OpenSea	*C12orf79*
8.56E-06	14	107078376	cg00715313	-0.46	0.035	OpenSea	
8.76E-06	6	146214507	cg08433435	-0.32	0.036	OpenSea	*SHPRH*
9.01E-06	20	54987140	cg24587601	0.32	0.037	OpenSea	*CASS4*
9.02E-06	14	70242852	cg14059534	0.39	0.037	OpenSea	*SLC10A1*
9.66E-06	19	16629806	cg14798382	-0.46	0.039	N_Shore	*CHERP*
9.71E-06	19	1822445	cg17380091	0.19	0.039	Island	*REXO1*
9.77E-06	17	71592079	cg24768077	0.28	0.039	OpenSea	*SDK2*
9.81E-06	7	78138408	cg17195693	-0.26	0.039	OpenSea	*MAGI2*
1E-05	1	27282675	cg19681352	0.45	0.040	N_Shelf	*C1orf172*
1.06E-05	4	55521081	cg16915285	0.30	0.042	N_Shelf	
1.12E-05	2	63711824	cg10178413	-0.21	0.044	OpenSea	*WDPCP*
1.12E-05	5	110848437	cg11890437	0.24	0.044	Island	*STARD4*
1.16E-05	3	101877174	cg22728989	-0.21	0.045	OpenSea	
1.18E-05	12	860520	cg25150924	0.19	0.045	N_Shore	

Chr: Chromosome. Pos: Position. Log FC: Log fold change.

Table S6. DMPs associated with Y (FDR < 0.05) adjusted for age, BMI, mother education, and estimated cell type proportions.

***p*-value**	**Chr**	**Pos**	**CpG**	**Log FC**	**FDR**	**Relation_to_Island**	**Gene**
3.84E-11	13	90045847	cg24712244	-0.27	0.000	OpenSea	
1.58E-09	3	138830878	cg14638147	0.41	0.001	OpenSea	*BPESC1*
2.71E-09	18	47123950	cg12490577	0.42	0.001	OpenSea	
5.78E-09	17	3704574	cg13984928	-0.40	0.001	OpenSea	*ITGAE*
8.29E-09	5	122947164	cg03722513	-0.24	0.001	OpenSea	*CSNK1G3*
9.41E-09	7	128697416	cg06396390	0.38	0.001	S_Shore	
1.50E-08	1	53207187	cg09509462	-0.34	0.002	OpenSea	*ZYG11B*
2.56E-08	20	36628932	cg01720774	0.36	0.002	OpenSea	*TTI1*
2.77E-08	12	113440283	cg24531077	0.44	0.002	OpenSea	*OAS2*
2.84E-08	12	51419973	cg04118903	-0.29	0.002	Island	*SLC11A2*
4.43E-08	11	31530876	cg15088759	0.45	0.002	N_Shore	*ELP4*
4.48E-08	10	111967167	cg21196958	0.47	0.002	N_Shore	*MXI1*
6.01E-08	17	2652918	cg09652807	-0.46	0.003	Island	*MIR1253*
6.09E-08	19	30862847	cg15977798	-0.22	0.003	N_Shelf	*ZNF536*
6.36E-08	2	75708900	cg17706091	0.34	0.003	OpenSea	
6.72E-08	10	17051623	cg20434599	0.53	0.003	OpenSea	*CUBN*
7.05E-08	10	12742170	cg01167366	0.87	0.003	OpenSea	*CAMK1D*
8.93E-08	17	49450892	cg02960148	0.34	0.003	OpenSea	
1.10E-07	10	47667303	cg26231261	0.34	0.004	OpenSea	*ANTXRL*
1.11E-07	16	19890592	cg01620602	0.39	0.004	OpenSea	*GPRC5B*
1.30E-07	1	161981053	cg27316795	0.65	0.004	OpenSea	*OLFML2B*
1.30E-07	1	241779250	cg03217228	0.62	0.004	OpenSea	*OPN3*
1.32E-07	6	166205748	cg13220290	0.31	0.004	OpenSea	
1.37E-07	8	144775130	cg05342816	0.38	0.004	N_Shore	*ZNF707*
1.48E-07	3	47563649	cg04507495	-0.43	0.004	OpenSea	
1.58E-07	8	1840794	cg24613789	0.39	0.004	OpenSea	*ARHGEF10*
1.62E-07	1	109582945	cg03677021	-0.41	0.004	N_Shore	*WDR47*
1.79E-07	5	93954434	cg11339965	0.38	0.004	Island	*ANKRD32*
1.81E-07	7	27155036	cg16406967	0.40	0.004	Island	*HOXA3*
1.97E-07	11	67829755	cg06724394	0.30	0.004	OpenSea	*CHKA*
1.99E-07	22	38541473	cg19871462	0.43	0.004	OpenSea	*PLA2G6*
2.02E-07	1	50883329	cg22711792	0.45	0.004	Island	*DMRTA2*
2.03E-07	6	97638598	cg15778867	-0.29	0.004	OpenSea	*MIR548H3*
2.23E-07	3	196050645	cg27088176	0.59	0.004	OpenSea	*TM4SF19*
2.43E-07	10	45098623	cg01146879	-0.24	0.005	OpenSea	
2.51E-07	1	224803364	cg09558034	0.51	0.005	N_Shore	*CNIH3*
2.56E-07	1	41960295	cg09548495	0.54	0.005	N_Shore	
2.67E-07	14	22217354	cg00208384	0.50	0.005	OpenSea	
2.70E-07	1	227585342	cg12245524	0.36	0.005	OpenSea	

p-value	Chr	Pos	CpG	Log FC	FDR	Relation_to_Island	Gene
2.71E-07	18	19799634	cg15651884	0.42	0.005	OpenSea	
2.94E-07	2	38831686	cg02565468	0.37	0.005	S_Shore	
3.05E-07	17	81037199	cg07377662	0.38	0.005	Island	*METRNL*
3.11E-07	2	233285289	cg13193840	0.49	0.005	Island	
3.14E-07	18	35101941	cg18032297	0.35	0.005	N_Shelf	*BRUNOL4*
3.32E-07	3	72701850	cg07127883	0.38	0.005	N_Shelf	
3.40E-07	14	76070515	cg10993988	-0.23	0.005	OpenSea	*MIR7641-2*
3.54E-07	9	136739643	cg09136786	0.33	0.005	OpenSea	*VAV2*
3.72E-07	5	39373418	cg02653030	0.37	0.005	OpenSea	*DAB2*
3.94E-07	8	1330464	cg03215535	0.63	0.005	S_Shelf	
4.02E-07	16	67380005	cg03956914	-0.23	0.005	OpenSea	*LRRC36*
4.40E-07	3	101877174	cg22728989	-0.20	0.006	OpenSea	
4.49E-07	1	91192798	cg14663264	0.36	0.006	Island	
5.07E-07	3	135969053	cg26010175	0.52	0.006	Island	*PCCB*
5.32E-07	14	70242852	cg14059534	0.36	0.007	OpenSea	*SLC10A1*
5.43E-07	1	31474920	cg02771649	0.56	0.007	OpenSea	*PUM1*
5.49E-07	3	14200360	cg08054515	0.26	0.007	OpenSea	*XPC*
5.83E-07	10	99052468	cg06456389	0.70	0.007	Island	*ARHGAP19*
5.95E-07	17	46894684	cg12027420	0.41	0.007	Island	*TTLL6*
6.06E-07	14	104400143	cg24270471	0.29	0.007	OpenSea	*TDRD9*
6.14E-07	7	99772832	cg13246281	0.29	0.007	N_Shore	*GPC2*
6.18E-07	6	29442830	cg10619365	0.37	0.007	OpenSea	
6.29E-07	11	14435856	cg05003364	-0.23	0.007	OpenSea	
6.30E-07	1	155829128	cg11545364	0.41	0.007	Island	*SYT11*
6.40E-07	4	157119935	cg11294620	0.23	0.007	OpenSea	
6.68E-07	10	104436518	cg27316828	0.55	0.007	OpenSea	*ARL3*
6.74E-07	2	45870883	cg08715805	0.49	0.007	Island	
7.24E-07	7	27155002	cg16748008	0.33	0.007	Island	*HOXA3*
7.29E-07	6	589274	cg14653814	0.18	0.007	OpenSea	*EXOC2*
7.46E-07	10	75012760	cg03037366	0.33	0.007	S_Shore	*MRPS16*
7.58E-07	16	87309686	cg05541311	0.45	0.007	OpenSea	
7.69E-07	16	2529732	cg26531633	0.27	0.007	S_Shelf	*TBC1D24*
7.76E-07	22	39899355	cg22773662	0.52	0.007	S_Shore	*MIEF1*
8.32E-07	11	115631394	cg25659317	0.49	0.008	S_Shore	*LINC00900*
8.47E-07	5	114763264	cg17570719	0.38	0.008	OpenSea	
8.84E-07	17	78822725	cg13945540	0.40	0.008	OpenSea	*RPTOR*
8.92E-07	8	110342178	cg27574309	-0.25	0.008	N_Shelf	*NUDCD1*
8.95E-07	2	242120289	cg13734948	0.46	0.008	OpenSea	*PPP1R7*
9.04E-07	19	3866347	cg19705859	0.30	0.008	N_Shelf	*ZFR2*
9.12E-07	18	6543922	cg01786044	0.36	0.008	OpenSea	*LINC01387*

p-value	Chr	Pos	CpG	Log FC	FDR	Relation_to_Island	Gene
9.37E-07	6	29794008	cg08708750	-0.33	0.008	N_Shore	*HLA-G*
1.02E-06	19	18946913	cg15482025	0.38	0.009	S_Shelf	*UPF1*
1.07E-06	7	51103766	cg06488615	-0.17	0.009	OpenSea	*COBL*
1.10E-06	20	18277898	cg18896550	-0.21	0.009	OpenSea	*ZNF133*
1.12E-06	6	6113358	cg19380199	-0.18	0.009	OpenSea	
1.12E-06	7	27155039	cg18680977	0.40	0.009	Island	*HOXA3*
1.17E-06	12	124121050	cg12017810	0.28	0.009	S_Shelf	*GTF2H3*
1.19E-06	5	172566090	cg25152909	0.34	0.009	OpenSea	*C5orf41*
1.22E-06	17	8815841	cg24251850	0.25	0.010	OpenSea	*PIK3R5*
1.23E-06	7	27154845	cg16644023	0.35	0.010	N_Shore	*HOXA3*
1.29E-06	17	57228640	cg02611741	-0.21	0.010	N_Shelf	*MIR301A*
1.31E-06	5	94891623	cg03977394	0.44	0.010	S_Shore	*ARSK*
1.33E-06	2	150571527	cg22274628	-0.21	0.010	OpenSea	*LOC101929231*
1.38E-06	16	89162377	cg06359492	0.30	0.010	S_Shore	*ACSF3*
1.41E-06	17	81018426	cg08040148	-0.43	0.010	S_Shelf	
1.49E-06	6	84446247	cg18166979	-0.26	0.011	OpenSea	
1.65E-06	16	103646	cg09969830	-0.20	0.012	Island	*SNRNP25*
1.66E-06	1	20864944	cg24604310	-0.23	0.012	OpenSea	
1.67E-06	17	71592079	cg24768077	0.26	0.012	OpenSea	*SDK2*
1.72E-06	8	27779092	cg11133658	0.46	0.012	Island	*SCARA5*
1.76E-06	9	35828874	cg01543404	0.35	0.012	Island	*TMEM8B*
1.84E-06	13	97422029	cg09122588	0.76	0.013	OpenSea	*HS6ST3*
1.85E-06	6	7469052	cg12753986	0.51	0.013	OpenSea	
1.95E-06	6	132722778	cg13640626	0.43	0.013	Island	*MOXD1*
1.97E-06	19	41945352	cg14054120	0.32	0.013	N_Shore	*ATP5SL*
1.98E-06	15	65068371	cg12725420	0.52	0.013	S_Shore	*RBPMS2*
2.01E-06	2	207139445	cg13788515	-0.18	0.013	Island	*ZDBF2*
2.12E-06	18	69503710	cg22773954	-0.28	0.014	OpenSea	
2.13E-06	16	56485455	cg14837652	0.32	0.014	Island	*NUDT21*
2.20E-06	3	137725933	cg16240260	-0.26	0.014	N_Shelf	*CLDN18*
2.27E-06	7	78138408	cg17195693	-0.23	0.014	OpenSea	*MAGI2*
2.29E-06	1	114212812	cg04141901	0.26	0.014	OpenSea	*MAGI3*
2.32E-06	15	76151117	cg11384517	0.39	0.014	OpenSea	*UBE2Q2*
2.34E-06	4	186541367	cg04764474	0.50	0.014	N_Shelf	*SORBS2*
2.40E-06	3	182591616	cg05002500	-0.17	0.014	OpenSea	*ATP11B*
2.41E-06	3	178055626	cg13609889	-0.22	0.014	OpenSea	
2.42E-06	9	134273620	cg10282135	0.21	0.014	S_Shelf	
2.42E-06	17	25619946	cg07564598	0.39	0.014	N_Shore	*WSB1*
2.57E-06	7	15105769	cg17867321	-0.18	0.015	OpenSea	
2.58E-06	8	6603212	cg06460618	0.32	0.015	OpenSea	*MIR4659B*

p-value	Chr	Pos	CpG	Log FC	FDR	Relation_to_Island	Gene
2.62E-06	11	134681766	cg16729283	0.34	0.015	OpenSea	
2.76E-06	6	31862388	cg08249097	-0.21	0.016	N_Shelf	*EHMT2*
2.81E-06	8	40059435	cg20660197	0.29	0.016	OpenSea	
2.88E-06	13	111907965	cg07073749	-0.23	0.016	S_Shore	*ARHGEF7*
2.92E-06	10	54784994	cg19096327	-0.28	0.016	OpenSea	
2.92E-06	17	14642137	cg05801153	0.35	0.016	OpenSea	
3.05E-06	12	40559300	cg01126347	-0.19	0.017	OpenSea	
3.08E-06	11	3735574	cg09537015	0.22	0.017	OpenSea	*NUP98*
3.16E-06	17	66286791	cg07407787	0.14	0.017	Island	*ARSG*
3.25E-06	7	128415071	cg09658621	0.36	0.018	OpenSea	*OPN1SW*
3.30E-06	8	75288417	cg00190393	-0.25	0.018	OpenSea	
3.31E-06	5	73463484	cg01195564	0.23	0.018	OpenSea	
3.31E-06	2	86161043	cg21065964	-0.16	0.018	OpenSea	
3.34E-06	12	76159482	cg24835883	0.32	0.018	OpenSea	
3.44E-06	11	95779454	cg25267808	0.37	0.018	OpenSea	*MAML2*
3.46E-06	12	98811404	cg23053746	0.27	0.018	OpenSea	
3.52E-06	19	6268276	cg08520648	0.32	0.018	N_Shelf	*MLLT1*
3.59E-06	2	39512317	cg21990552	-0.25	0.018	OpenSea	*MAP4K3*
3.63E-06	11	1945648	cg11654118	0.32	0.018	N_Shore	*TNNT3*
3.64E-06	9	96272718	cg00688269	0.26	0.018	OpenSea	*FAM120A*
3.65E-06	8	53556400	cg08986465	-0.28	0.018	OpenSea	*RB1CC1*
3.67E-06	16	74971138	cg02313132	0.51	0.018	OpenSea	*WDR59*
4.03E-06	14	58619219	cg18463820	0.43	0.020	Island	*C14orf37*
4.41E-06	8	81398656	cg22380165	0.44	0.022	Island	*ZBTB10*
4.56E-06	1	43123748	cg20988549	0.30	0.022	N_Shore	*PPIH*
4.62E-06	21	43324038	cg02590715	0.35	0.022	N_Shelf	*C2CD2*
4.63E-06	19	36139674	cg16053099	0.19	0.022	S_Shelf	*COX6B1*
4.80E-06	3	56792794	cg27553488	0.21	0.023	OpenSea	*ARHGEF3*
4.91E-06	10	90774324	cg23169494	-0.24	0.023	OpenSea	*FAS*
4.98E-06	11	7108907	cg21241829	-0.18	0.023	N_Shore	*RBMXL2*
5.06E-06	17	35974843	cg02009728	-0.24	0.024	OpenSea	*DDX52*
5.12E-06	15	72853731	cg05549853	-0.22	0.024	OpenSea	*ARIH1*
5.23E-06	8	103819572	cg03547517	0.70	0.024	S_Shore	
5.33E-06	5	56246733	cg08288226	0.26	0.025	Island	*MIER3*
5.41E-06	14	21029481	cg22768584	-0.18	0.025	OpenSea	*RNASE9*
5.45E-06	12	133198653	cg21162817	0.36	0.025	S_Shelf	*P2RX2*
5.56E-06	1	155036577	cg23008177	0.29	0.025	Island	*EFNA4*
5.65E-06	6	108930710	cg26165197	0.22	0.025	OpenSea	*FOXO3*
5.66E-06	9	138145731	cg13818014	-0.56	0.025	OpenSea	
6.20E-06	6	10690667	cg07606551	0.22	0.027	N_Shelf	*C6orf52*

p-value	Chr	Pos	CpG	Log FC	FDR	Relation_to_Island	Gene
6.29E-06	15	93426986	cg02276914	0.15	0.028	S_Shore	*LINC01578*
6.52E-06	2	18705868	cg04497514	0.31	0.029	OpenSea	
6.56E-06	14	90399131	cg09993641	-0.22	0.029	OpenSea	*EFCAB11*
6.69E-06	6	146214507	cg08433435	-0.28	0.029	OpenSea	*SHPRH*
6.89E-06	8	31883201	cg20240230	-0.30	0.030	OpenSea	*NRG1-IT1*
7.04E-06	5	110848437	cg11890437	0.21	0.030	Island	*STARD4*
7.05E-06	10	180329	cg02867818	0.36	0.030	N_Shore	*ZMYND11*
7.09E-06	15	79296424	cg06376992	-0.20	0.030	OpenSea	*RASGRF1*
7.23E-06	2	102026463	cg07099118	-0.24	0.030	OpenSea	*RFX8*
7.36E-06	3	78079111	cg10728960	0.45	0.031	OpenSea	
7.73E-06	1	158174399	cg07377195	-0.34	0.032	OpenSea	
7.79E-06	13	95846842	cg00241432	0.34	0.032	OpenSea	*ABCC4*
8.36E-06	18	44373583	cg08113537	0.32	0.034	OpenSea	
8.71E-06	19	49994372	cg09372739	0.34	0.036	S_Shelf	*SNORD35A*
9.04E-06	8	28535162	cg12221428	0.39	0.037	OpenSea	
9.07E-06	18	46621257	cg20465758	-0.16	0.037	OpenSea	*DYM*
9.18E-06	6	30904981	cg08487909	0.17	0.037	OpenSea	
9.47E-06	19	44278274	cg03731131	0.18	0.038	Island	*KCNN4*
9.62E-06	1	214159131	cg06623935	-0.27	0.038	N_Shore	
9.66E-06	3	81792982	cg02639218	0.25	0.038	OpenSea	*GBE1*
9.77E-06	4	78774513	cg16526120	0.46	0.038	OpenSea	
1.02E-05	6	33281712	cg23381664	0.32	0.040	Island	*TAPBP*
1.02E-05	1	112533012	cg21348554	0.20	0.040	Island	*KCND3*
1.03E-05	11	31443485	cg27180150	-0.20	0.040	OpenSea	*DNAJC24*
1.05E-05	7	27154914	cg00318947	0.27	0.040	N_Shore	*HOXA3*
1.07E-05	2	231068094	cg05408985	0.30	0.040	OpenSea	*SP110*
1.09E-05	5	121677927	cg01790628	0.30	0.041	OpenSea	*SNCAIP*
1.10E-05	2	109204687	cg02385799	0.38	0.041	OpenSea	*LIMS1*
1.11E-05	2	1232428	cg16653700	-0.24	0.041	OpenSea	*SNTG2*
1.13E-05	1	1957711	cg10837110	-0.42	0.042	N_Shore	*GABRD*
1.14E-05	12	63479872	cg04774139	-0.19	0.042	OpenSea	
1.14E-05	6	36842844	cg26222229	0.15	0.042	Island	*PPIL1*
1.15E-05	19	44285426	cg12787665	-0.20	0.042	OpenSea	*KCNN4*
1.15E-05	12	53440686	cg20149600	0.24	0.042	Island	*TENC1*
1.15E-05	7	27155173	cg04351734	0.32	0.042	Island	*HOXA3*
1.15E-05	12	392537	cg20024259	-0.14	0.042	OpenSea	*KDM5A*
1.17E-05	10	112466738	cg13331591	0.27	0.042	OpenSea	*RBM20*
1.19E-05	6	108977419	cg09546902	0.34	0.042	OpenSea	*FOXO3*
1.21E-05	14	107078376	cg00715313	-0.38	0.043	OpenSea	
1.22E-05	14	74680453	cg12779982	0.39	0.043	N_Shelf	

p-value	Chr	Pos	CpG	Log FC	FDR	Relation_to_Island	Gene
1.23E-05	14	87262857	cg21871338	0.39	0.043	OpenSea	
1.29E-05	16	84792860	cg01430302	0.24	0.045	OpenSea	*USP10*
1.29E-05	8	38872513	cg04026002	-0.27	0.045	OpenSea	*ADAM9*
1.33E-05	6	72244099	cg18362986	-0.14	0.046	OpenSea	
1.37E-05	18	13375540	cg21243597	-0.13	0.047	OpenSea	*C18orf1*
1.43E-05	2	170397488	cg13850024	-0.23	0.049	OpenSea	*FASTKD1*
1.46E-05	12	125534043	cg13938881	0.50	0.050	N_Shore	
1.47E-05	12	104378097	cg27454347	-0.19	0.050	OpenSea	*TDG*
1.48E-05	5	150936730	cg06071063	0.26	0.050	OpenSea	*FAT2*
1.49E-05	20	46792569	cg01135693	-0.14	0.050	OpenSea	

Chr: Chromosome. Pos: Position. Log FC: Log fold change.

Table S7. DMPs associated with Sn (FDR < 0.05) adjusted for age, BMI, mother education, and estimated cell type proportions.

***p*-value**	**Chr**	**Pos**	**CpG**	**Log FC**	**FDR**	**Relation_to_Island**	**Gene**
4.69E-09	7	27155173	cg04351734	0.32	0.002	Island	*HOXA3*
4.87E-09	7	27155036	cg16406967	0.36	0.002	Island	*HOXA3*
7.11E-09	7	128415071	cg09658621	0.35	0.002	OpenSea	*OPN1SW*
9.24E-09	21	43324038	cg02590715	0.34	0.002	N_Shelf	*C2CD2*
1.33E-08	5	39373418	cg02653030	0.33	0.002	OpenSea	*DAB2*
1.73E-08	3	44912362	cg02449689	0.29	0.002	OpenSea	
1.81E-08	17	49450892	cg02960148	0.29	0.002	OpenSea	
1.82E-08	7	27155002	cg16748008	0.30	0.002	Island	*HOXA3*
2.96E-08	7	27155039	cg18680977	0.36	0.002	Island	*HOXA3*
3.26E-08	18	19799634	cg15651884	0.37	0.002	OpenSea	
3.29E-08	17	78822725	cg13945540	0.36	0.002	OpenSea	*RPTOR*
3.77E-08	10	17051623	cg20434599	0.45	0.002	OpenSea	*CUBN*
3.96E-08	16	89162377	cg06359492	0.27	0.002	S_Shore	*ACSF3*
3.99E-08	19	18946913	cg15482025	0.34	0.002	S_Shelf	*UPF1*
4.54E-08	16	19890592	cg01620602	0.34	0.002	OpenSea	*GPRC5B*
5.09E-08	1	224803364	cg09558034	0.44	0.002	N_Shore	*CNIH3*
5.18E-08	5	172566090	cg25152909	0.31	0.002	OpenSea	*C5orf41*
6.01E-08	12	76159482	cg24835883	0.29	0.002	OpenSea	
6.21E-08	8	81398656	cg22380165	0.41	0.002	Island	*ZBTB10*
6.39E-08	6	29442830	cg10619365	0.32	0.002	OpenSea	
7.47E-08	10	99052468	cg06456389	0.62	0.003	Island	*ARHGAP19*
7.67E-08	10	104436518	cg27316828	0.48	0.003	OpenSea	*ARL3*
9.37E-08	8	144775130	cg05342816	0.32	0.003	N_Shore	*ZNF707*
1.10E-07	10	72647938	cg25757740	0.36	0.003	Island	*PCBD1*
1.12E-07	9	35828874	cg01543404	0.32	0.003	Island	*TMEM8B*
1.15E-07	18	69503710	cg22773954	-0.26	0.003	OpenSea	
1.15E-07	17	25619946	cg07564598	0.36	0.003	N_Shore	*WSB1*
1.19E-07	10	47667303	cg26231261	0.29	0.003	OpenSea	*ANTXRL*
1.37E-07	2	18705868	cg04497514	0.29	0.003	OpenSea	
1.43E-07	4	186541367	cg04764474	0.45	0.003	N_Shelf	*SORBS2*
1.54E-07	1	31474920	cg02771649	0.49	0.003	OpenSea	*PUM1*
1.59E-07	14	70242852	cg14059534	0.31	0.003	OpenSea	*SLC10A1*
1.70E-07	14	102176671	cg19730972	-0.35	0.004	S_Shelf	
1.85E-07	22	38541473	cg19871462	0.36	0.004	OpenSea	*PLA2G6*
1.94E-07	2	170397488	cg13850024	-0.22	0.004	OpenSea	*FASTKD1*
2.01E-07	16	85317070	cg27079934	0.29	0.004	N_Shore	*LINC00311*
2.06E-07	17	2652918	cg09652807	-0.38	0.004	Island	*MIR1253*
2.09E-07	18	47123950	cg12490577	0.32	0.004	OpenSea	
2.18E-07	17	3704574	cg13984928	-0.31	0.004	OpenSea	*ITGAE*

p-value	Chr	Pos	CpG	Log FC	FDR	Relation_to_Island	Gene
2.23E-07	17	81018426	cg08040148	-0.37	0.004	S_Shelf	
2.25E-07	1	41960295	cg09548495	0.45	0.004	N_Shore	
2.47E-07	1	63791999	cg06198384	0.41	0.004	S_Shore	
2.59E-07	1	241779250	cg03217228	0.51	0.004	OpenSea	*OPN3*
2.6E2-07	7	128697416	cg06396390	0.30	0.004	S_Shore	
2.67E-07	1	161981053	cg27316795	0.53	0.004	OpenSea	*OLFML2B*
2.80E-07	12	98811404	cg23053746	0.24	0.004	OpenSea	
2.84E-07	19	3866347	cg19705859	0.26	0.004	N_Shelf	*ZFR2*
3.06E-07	6	142407908	cg01477493	-0.18	0.004	N_Shore	*NMBR*
3.07E-07	11	67829755	cg06724394	0.25	0.004	OpenSea	*CHKA*
3.18E-07	19	6268276	cg08520648	0.29	0.004	N_Shelf	*MLLT1*
3.20E-07	6	132722778	cg13640626	0.38	0.004	Island	*MOXD1*
3.31E-07	2	231068094	cg05408985	0.28	0.004	OpenSea	*SP110*
3.33E-07	15	65068371	cg12725420	0.45	0.004	S_Shore	*RBPMS2*
3.45E-07	2	38831686	cg02565468	0.31	0.004	S_Shore	
3.47E-07	2	102026463	cg07099118	-0.22	0.004	OpenSea	*RFX8*
3.55E-07	15	76151117	cg11384517	0.34	0.004	OpenSea	*UBE2Q2*
3.57E-07	18	46621257	cg20465758	-0.15	0.004	OpenSea	*DYM*
3.58E-07	8	54149006	cg10864028	0.22	0.004	OpenSea	*OPRK1*
3.66E-07	17	8815841	cg24251850	0.21	0.004	OpenSea	*PIK3R5*
3.84E-07	1	234211980	cg08885541	0.28	0.004	OpenSea	*SLC35F3*
3.84E-07	8	27779092	cg11133658	0.40	0.004	Island	*SCARA5*
4.01E-07	4	3533876	cg16565635	-0.30	0.004	Island	*LRPAP1*
4.07E-07	12	51419973	cg04118903	-0.23	0.004	Island	*SLC11A2*
4.07E-07	7	27154845	cg16644023	0.30	0.004	N_Shore	*HOXA3*
4.10E-07	17	71592079	cg24768077	0.22	0.004	OpenSea	*SDK2*
4.18E-07	6	150329845	cg27001715	-0.66	0.004	S_Shelf	
4.39E-07	1	155036577	cg23008177	0.26	0.005	Island	*EFNA4*
4.52E-07	16	732232	cg17546868	0.25	0.005	Island	*STUB1*
4.71E-07	3	78079111	cg10728960	0.40	0.005	OpenSea	
4.74E-07	5	157702593	cg18702684	0.21	0.005	OpenSea	
4.89E-07	12	133198653	cg21162817	0.32	0.005	S_Shelf	*P2RX2*
5.27E-07	5	114763264	cg17570719	0.32	0.005	OpenSea	
5.73E-07	4	55338905	cg21813288	0.20	0.006	OpenSea	
5.79E-07	1	109582945	cg03677021	-0.33	0.006	N_Shore	*WDR47*
5.84E-07	8	1330464	cg03215535	0.52	0.006	S_Shelf	
6.15E-07	10	180329	cg02867818	0.33	0.006	N_Shore	*ZMYND11*
6.15E-07	1	65619463	cg17537030	0.24	0.006	OpenSea	*AK4*
6.19E-07	2	109204687	cg02385799	0.34	0.006	OpenSea	*LIMS1*
6.21E-07	2	52524614	cg00633008	0.22	0.006	OpenSea	

p-value	Chr	Pos	CpG	Log FC	FDR	Relation_to_Island	Gene
6.23E-07	4	157119935	cg11294620	0.19	0.006	OpenSea	
6.55E-07	1	158174399	cg07377195	-0.30	0.006	OpenSea	
6.67E-07	3	178055626	cg13609889	-0.19	0.006	OpenSea	
7.54E-07	2	45870883	cg08715805	0.41	0.006	Island	
7.92E-07	4	3533922	cg23528723	-0.27	0.007	Island	*LRPAP1*
8.01E-07	12	113440283	cg24531077	0.34	0.007	OpenSea	*OAS2*
8.28E-07	2	137950714	cg17180863	0.84	0.007	OpenSea	*THSD7B*
9.34E-07	11	3735574	cg09537015	0.19	0.008	OpenSea	*NUP98*
9.57E-07	6	166205748	cg13220290	0.25	0.008	OpenSea	
9.60E-07	3	14200360	cg08054515	0.22	0.008	OpenSea	*XPC*
1.01E-06	11	31443485	cg27180150	-0.18	0.008	OpenSea	*DNAJC24*
1.09E-06	3	101877174	cg22728989	-0.16	0.008	OpenSea	
1.10E-06	3	196050645	cg27088176	0.47	0.008	OpenSea	*TM4SF19*
1.16E-06	2	233285289	cg13193840	0.40	0.009	Island	
1.20E-06	3	151596028	cg13634423	0.24	0.009	OpenSea	*SUCNR1*
1.20E-06	16	87309686	cg05541311	0.37	0.009	OpenSea	
1.22E-06	10	112466738	cg13331591	0.25	0.009	OpenSea	*RBM20*
1.25E-06	9	136739643	cg09136786	0.27	0.009	OpenSea	*VAV2*
1.33E-06	5	118332221	cg17136442	0.34	0.009	OpenSea	
1.36E-06	7	27154911	cg15982700	0.28	0.010	N_Shore	*HOXA3*
1.43E-06	5	150936730	cg06071063	0.24	0.010	OpenSea	*FAT2*
1.47E-06	6	108930710	cg26165197	0.19	0.010	OpenSea	*FOXO3*
1.52E-06	1	37555561	cg18179395	0.15	0.010	OpenSea	
1.56E-06	7	99772832	cg13246281	0.23	0.011	N_Shore	*GPC2*
1.66E-06	20	56135029	cg10278454	0.23	0.011	OpenSea	*PCK1*
1.68E-06	7	78138408	cg17195693	-0.19	0.011	OpenSea	*MAGI2*
1.88E-06	1	227585342	cg12245524	0.29	0.012	OpenSea	
2.16E-06	7	161985	cg00846539	-0.11	0.014	S_Shore	
2.28E-06	1	53207187	cg09509462	-0.25	0.015	OpenSea	*ZYG11B*
2.46E-06	12	119616484	cg05831308	0.32	0.016	OpenSea	*HSPB8*
2.52E-06	14	58619219	cg18463820	0.36	0.016	Island	*C14orf37*
2.60E-06	6	10690667	cg07606551	0.19	0.016	N_Shelf	*C6orf52*
2.63E-06	13	95846842	cg00241432	0.29	0.016	OpenSea	*ABCC4*
2.75E-06	1	1957711	cg10837110	-0.37	0.017	N_Shore	*GABRD*
2.84E-06	6	33807279	cg12241963	0.15	0.017	OpenSea	
2.91E-06	17	1080239	cg16050472	-0.13	0.018	N_Shelf	*ABR*
2.97E-06	3	130649266	cg10774974	-0.16	0.018	OpenSea	*ATP2C1*
3.05E-06	10	112404754	cg06143897	0.22	0.018	S_Shore	*RBM20*
3.06E-06	9	37780478	cg13441107	0.16	0.018	OpenSea	*EXOSC3*
3.11E-06	12	92384227	cg08921760	-0.20	0.018	OpenSea	*C12orf79*

p-value	Chr	Pos	CpG	Log FC	FDR	Relation_to_Island	Gene
3.26E-06	10	75012760	cg03037366	0.27	0.019	S_Shore	*MRPS16*
3.37E-06	12	124121050	cg12017810	0.23	0.019	S_Shelf	*GTF2H3*
3.42E-06	16	103646	cg09969830	-0.16	0.020	Island	*SNRNP25*
3.51E-06	1	155829128	cg11545364	0.33	0.020	Island	*SYT11*
3.54E-06	14	89821276	cg13397551	-0.29	0.020	OpenSea	*FOXN3*
3.70E-06	7	27154720	cg21556281	0.25	0.021	N_Shore	*HOXA3*
3.75E-06	1	114212812	cg04141901	0.22	0.021	OpenSea	*MAGI3*
3.84E-06	11	31530876	cg15088759	0.34	0.021	N_Shore	*ELP4*
3.89E-06	7	27154914	cg00318947	0.23	0.021	N_Shore	*HOXA3*
3.92E-06	1	192597225	cg24700374	-0.21	0.021	OpenSea	
3.93E-06	17	14642137	cg05801153	0.29	0.021	OpenSea	
3.93E-06	3	47563649	cg04507495	-0.33	0.021	OpenSea	
3.94E-06	19	1822445	cg17380091	0.14	0.021	Island	*REXO1*
3.96E-06	3	81792982	cg02639218	0.22	0.021	OpenSea	*GBE1*
3.99E-06	11	134681766	cg16729283	0.28	0.021	OpenSea	
4.03E-06	12	63479872	cg04774139	-0.17	0.021	OpenSea	
4.22E-06	6	31647562	cg21561281	0.26	0.022	N_Shore	*LY6G5C*
4.22E-06	20	62369445	cg01176363	-0.46	0.022	Island	*LIME1*
4.24E-06	6	108977419	cg09546902	0.29	0.022	OpenSea	*FOXO3*
4.32E-06	2	75708900	cg17706091	0.25	0.022	OpenSea	
4.34E-06	10	12742170	cg01167366	0.65	0.022	OpenSea	*CAMK1D*
4.35E-06	2	208394989	cg05727155	0.12	0.022	Island	*CREB1*
4.38E-06	8	28535162	cg12221428	0.33	0.022	OpenSea	
4.44E-06	1	183440077	cg10164018	0.35	0.022	N_Shore	*SMG7*
4.49E-06	7	135662562	cg21537187	0.70	0.022	S_Shore	*MTPN*
5.49E-06	21	45622373	cg11113661	0.14	0.027	Island	
5.80E-06	17	599829	cg12281099	0.30	0.028	OpenSea	*VPS53*
6.04E-06	22	30952581	cg24516286	0.18	0.029	S_Shore	*GAL3ST1*
6.21E-06	11	58724252	cg22133378	0.25	0.029	OpenSea	*GLYATL1*
6.47E-06	14	87262857	cg21871338	0.33	0.030	OpenSea	
6.49E-06	4	77120189	cg13524401	0.14	0.030	OpenSea	*SCARB2*
6.57E-06	16	67380005	cg03956914	-0.18	0.030	OpenSea	*LRRC36*
7.12E-06	1	41923683	cg02327542	0.11	0.033	OpenSea	
7.20E-06	12	92836720	cg05914217	0.24	0.033	OpenSea	
7.24E-06	1	158978812	cg04805131	-0.22	0.033	OpenSea	*IFI16*
7.34E-06	11	95779454	cg25267808	0.30	0.033	OpenSea	*MAML2*
7.46E-06	18	44373583	cg08113537	0.27	0.033	OpenSea	
7.75E-06	8	103819572	cg03547517	0.57	0.034	S_Shore	
7.75E-06	18	6543922	cg01786044	0.28	0.034	OpenSea	*LINC01387*
7.84E-06	1	40946609	cg00557440	0.19	0.034	S_Shelf	*ZNF642*

p-value	Chr	Pos	CpG	Log FC	FDR	Relation_to_Island	Gene
7.97E-06	9	80004560	cg08595278	-0.28	0.034	OpenSea	*VPS13A*
8.02E-06	2	179315654	cg17833257	0.27	0.034	Island	*DFNB59*
8.65E-06	17	46894684	cg12027420	0.32	0.037	Island	*TTLL6*
8.83E-06	4	148671546	cg11745567	0.28	0.038	OpenSea	*ARHGAP10*
8.95E-06	5	73463484	cg01195564	0.18	0.038	OpenSea	
8.97E-06	10	124905786	cg16361966	-0.17	0.038	Island	
9.02E-06	8	6603212	cg06460618	0.25	0.038	OpenSea	*MIR4659B*
9.26E-06	3	132759693	cg17566608	-0.22	0.038	S_Shore	*TMEM108*
9.70E-06	8	59490544	cg15864152	-0.27	0.040	OpenSea	*SDCBP*
9.86E-06	6	17988937	cg08248579	-0.40	0.040	S_Shore	*KIF13A*
9.87E-06	1	57320050	cg12264060	0.21	0.040	OpenSea	*C8A*
9.88E-06	6	589274	cg14653814	0.14	0.040	OpenSea	*EXOC2*
1.05E-05	2	73964144	cg07852972	0.23	0.042	N_Shore	*TPRKB*
1.05E-05	3	135969053	cg26010175	0.39	0.042	Island	*PCCB*
1.05E-05	8	1840794	cg24613789	0.29	0.042	OpenSea	*ARHGEF10*
1.09E-05	1	4107738	cg05079161	0.13	0.043	OpenSea	
1.09E-05	18	68695988	cg27532283	0.25	0.043	OpenSea	
1.11E-05	11	115631394	cg25659317	0.38	0.044	S_Shore	*LINC00900*
1.12E-05	6	66804830	cg22034507	-0.22	0.044	Island	
1.15E-05	13	111907965	cg07073749	-0.18	0.045	S_Shore	*ARHGEF7*
1.18E-05	13	74512388	cg18662671	0.21	0.046	OpenSea	*KLF12*
1.21E-05	2	242120289	cg13734948	0.36	0.046	OpenSea	*PPP1R7*
1.24E-05	20	48774608	cg16427387	-0.09	0.047	S_Shelf	
1.25E-05	3	30936070	cg18005693	-0.32	0.047	Island	*GADL1*
1.29E-05	14	103932525	cg21047303	-0.20	0.049	OpenSea	*MARK3*
1.30E-05	1	91192798	cg14663264	0.27	0.049	Island	
1.33E-05	7	98642053	cg22045105	-0.18	0.049	OpenSea	*SMURF1*
1.33E-05	3	42726082	cg22545174	0.27	0.049	N_Shore	*KLHL40*
1.34E-05	2	113341685	cg11317677	0.25	0.050	Island	*CHCHD5*
1.35E-05	7	138818391	cg01351421	0.15	0.050	Island	*TTC26*

Chr: Chromosome. Pos: Position. Log FC: Log fold change.

Table S8. DMPs associated with Ba (FDR < 0.05) adjusted for age, BMI, mother education, and estimated cell type proportions.

***p*-value**	**Chr**	**Pos**	**CpG**	**Log FC**	**FDR**	**Relation_to_Island**	**Gene**
5.55E-09	17	2652918	cg09652807	-0.35	0.004	Island	*MIR1253*
1.76E-08	7	27155036	cg16406967	0.30	0.005	Island	*HOXA3*
2.80E-08	7	27155039	cg18680977	0.31	0.005	Island	*HOXA3*
3.21E-08	7	27155173	cg04351734	0.27	0.005	Island	*HOXA3*
3.72E-08	7	27155002	cg16748008	0.26	0.005	Island	*HOXA3*
5.54E-08	15	76151117	cg11384517	0.31	0.007	OpenSea	*UBE2Q2*
9.51E-08	2	38831686	cg02565468	0.27	0.008	S_Shore	
1.05E-07	7	27154845	cg16644023	0.27	0.008	N_Shore	*HOXA3*
1.23E-07	10	47667303	cg26231261	0.25	0.009	OpenSea	*ANTXRL*
1.58E-07	16	19890592	cg01620602	0.28	0.009	OpenSea	*GPRC5B*
1.80E-07	8	144775130	cg05342816	0.27	0.009	N_Shore	*ZNF707*
1.97E-07	1	161981053	cg27316795	0.46	0.009	OpenSea	*OLFML2B*
2.04E-07	1	53207187	cg09509462	-0.23	0.009	OpenSea	*ZYG11B*
2.05E-07	17	78822725	cg13945540	0.30	0.009	OpenSea	*RPTOR*
2.11E-07	19	18946913	cg15482025	0.28	0.009	S_Shelf	*UPF1*
2.15E-07	19	6268276	cg08520648	0.25	0.009	N_Shelf	*MLLT1*
2.27E-07	12	76159482	cg24835883	0.24	0.009	OpenSea	
2.68E-07	18	47123950	cg12490577	0.27	0.009	OpenSea	
2.69E-07	7	128697416	cg06396390	0.26	0.009	S_Shore	
3.25E-07	21	43324038	cg02590715	0.27	0.010	N_Shelf	*C2CD2*
3.34E-07	5	114763264	cg17570719	0.28	0.010	OpenSea	
3.65E-07	18	69503710	cg22773954	-0.21	0.011	OpenSea	
3.86E-07	2	75708900	cg17706091	0.23	0.011	OpenSea	
3.89E-07	18	19799634	cg15651884	0.30	0.011	OpenSea	
4.06E-07	21	37616659	cg19478280	0.33	0.011	N_Shore	*DOPEY2*
4.59E-07	7	128415071	cg09658621	0.28	0.011	OpenSea	*OPN1SW*
4.63E-07	1	155036577	cg23008177	0.23	0.011	Island	*EFNA4*
4.69E-07	10	17051623	cg20434599	0.36	0.011	OpenSea	*CUBN*
4.82E-07	17	3704574	cg13984928	-0.26	0.011	OpenSea	*ITGAE*
5.99E-07	17	49450892	cg02960148	0.23	0.013	OpenSea	
7.14E-07	12	51419973	cg04118903	-0.19	0.015	Island	*SLC11A2*
7.27E-07	12	124121050	cg12017810	0.21	0.015	S_Shelf	*GTF2H3*
8.03E-07	2	18705868	cg04497514	0.24	0.016	OpenSea	
8.51E-07	22	38541473	cg19871462	0.30	0.017	OpenSea	*PLA2G6*
9.17E-07	3	47563649	cg04507495	-0.30	0.018	OpenSea	
1.02E-06	5	172566090	cg25152909	0.25	0.018	OpenSea	*C5orf41*
1.07E-06	18	44373583	cg08113537	0.24	0.018	OpenSea	
1.08E-06	4	100003288	cg23372626	0.20	0.018	OpenSea	*ADH5*
1.08E-06	19	3866347	cg19705859	0.21	0.018	N_Shelf	*ZFR2*

p-value	Chr	Pos	CpG	Log FC	FDR	Relation_to_Island	Gene
1.11E-06	5	39373418	cg02653030	0.26	0.018	OpenSea	*DAB2*
1.13E-06	2	233285289	cg13193840	0.34	0.018	Island	
1.15E-06	6	29442830	cg10619365	0.26	0.018	OpenSea	
1.24E-06	15	65068371	cg12725420	0.38	0.019	S_Shore	*RBPMS2*
1.25E-06	10	99052468	cg06456389	0.50	0.019	Island	*ARHGAP19*
1.30E-06	12	133198653	cg21162817	0.27	0.019	S_Shelf	*P2RX2*
1.31E-06	11	67829755	cg06724394	0.21	0.019	OpenSea	*CHKA*
1.47E-06	8	1330464	cg03215535	0.44	0.021	S_Shelf	
1.60E-06	13	90045847	cg24712244	-0.16	0.022	OpenSea	
1.62E-06	1	224803364	cg09558034	0.35	0.022	N_Shore	*CNIH3*
1.68E-06	1	41960295	cg09548495	0.37	0.023	N_Shore	
1.77E-06	13	95846842	cg00241432	0.25	0.023	OpenSea	*ABCC4*
1.83E-06	1	114212812	cg04141901	0.19	0.023	OpenSea	*MAGI3*
1.84E-06	14	70242852	cg14059534	0.25	0.023	OpenSea	*SLC10A1*
1.87E-06	3	44912362	cg02449689	0.22	0.023	OpenSea	
1.90E-06	1	241779250	cg03217228	0.42	0.023	OpenSea	*OPN3*
1.95E-06	3	72701850	cg07127883	0.26	0.024	N_Shelf	
2.05E-06	6	108930710	cg26165197	0.17	0.025	OpenSea	*FOXO3*
2.22E-06	9	136739643	cg09136786	0.23	0.026	OpenSea	*VAV2*
2.25E-06	14	36677210	cg15044055	0.15	0.026	OpenSea	
2.32E-06	6	12595019	cg16465027	0.42	0.026	OpenSea	
2.32E-06	4	8124699	cg00624710	0.21	0.026	OpenSea	*ABLIM2*
2.36E-06	1	31474920	cg02771649	0.39	0.026	OpenSea	*PUM1*
2.40E-06	11	134681766	cg16729283	0.24	0.026	OpenSea	
2.41E-06	3	14200360	cg08054515	0.18	0.026	OpenSea	*XPC*
2.45E-06	11	31443485	cg27180150	-0.15	0.026	OpenSea	*DNAJC24*
2.62E-06	20	36628932	cg01720774	0.23	0.027	OpenSea	*TTI1*
2.64E-06	10	104436518	cg27316828	0.38	0.027	OpenSea	*ARL3*
2.71E-06	2	231068094	cg05408985	0.23	0.027	OpenSea	*SP110*
2.85E-06	4	186541367	cg04764474	0.36	0.028	N_Shelf	*SORBS2*
3.01E-06	5	73463484	cg01195564	0.16	0.029	OpenSea	
3.03E-06	16	103646	cg09969830	-0.14	0.029	Island	*SNRNP25*
3.17E-06	12	63479872	cg04774139	-0.15	0.030	OpenSea	
3.23E-06	3	36422209	cg26615813	-0.35	0.030	Island	*STAC*
3.26E-06	3	196050645	cg27088176	0.39	0.030	OpenSea	*TM4SF19*
3.37E-06	16	89162377	cg06359492	0.21	0.031	S_Shore	*ACSF3*
3.49E-06	4	55338905	cg21813288	0.16	0.031	OpenSea	
3.50E-06	3	33131138	cg20024393	-0.11	0.031	OpenSea	*GLB1*
3.76E-06	11	3735574	cg09537015	0.16	0.033	OpenSea	*NUP98*
3.90E-06	3	132759693	cg17566608	-0.19	0.034	S_Shore	*TMEM108*

p-value	Chr	Pos	CpG	Log FC	FDR	Relation_to_Island	Gene
4.06E-06	2	45870883	cg08715805	0.33	0.035	Island	
4.14E-06	5	6868554	cg09731841	0.10	0.035	OpenSea	
4.15E-06	8	31883201	cg20240230	-0.22	0.035	OpenSea	*NRG1-IT1*
4.36E-06	14	107078376	cg00715313	-0.29	0.036	OpenSea	
4.41E-06	1	204587945	cg20382344	-0.19	0.036	OpenSea	*LRRN2*
4.59E-06	9	35828874	cg01543404	0.25	0.037	Island	*TMEM8B*
4.73E-06	1	18711973	cg09852604	-0.14	0.038	OpenSea	
4.90E-06	9	37780478	cg13441107	0.13	0.039	OpenSea	*EXOSC3*
4.97E-06	6	31647562	cg21561281	0.22	0.039	N_Shore	*LY6G5C*
5.17E-06	3	101877174	cg22728989	-0.13	0.040	OpenSea	
5.54E-06	17	1080239	cg16050472	-0.11	0.042	N_Shelf	*ABR*
5.59E-06	2	242120289	cg13734948	0.32	0.042	OpenSea	*PPP1R7*
5.60E-06	1	214159131	cg06623935	-0.20	0.042	N_Shore	
5.77E-06	12	113440283	cg24531077	0.27	0.043	OpenSea	*OAS2*
5.83E-06	10	12742170	cg01167366	0.55	0.043	OpenSea	*CAMK1D*
6.34E-06	8	27779092	cg11133658	0.32	0.046	Island	*SCARA5*
6.51E-06	17	48473757	cg20508508	-0.10	0.047	N_Shore	*LRRC59*
6.57E-06	12	98811404	cg23053746	0.19	0.047	OpenSea	
6.68E-06	7	27154911	cg15982700	0.23	0.047	N_Shore	*HOXA3*
6.73E-06	7	99772832	cg13246281	0.19	0.047	N_Shore	*GPC2*
6.84E-06	2	207139445	cg13788515	-0.12	0.047	Island	*ZDBF2*
7.12E-06	17	81018426	cg08040148	-0.29	0.048	S_Shelf	
7.22E-06	1	158978812	cg04805131	-0.19	0.048	OpenSea	*IFI16*
7.39E-06	11	26492697	cg27042693	-0.17	0.049	OpenSea	*ANO3*
7.47E-06	6	10690667	cg07606551	0.16	0.049	N_Shelf	*C6orf52*

Chr: Chromosome. Pos: Position. Log FC: Log fold change.
